# DTI-ALPS and subcortical structural-functional coupling mediate the impact of sleep quality on working memory in insomnia disorder

**DOI:** 10.1017/S0033291726104188

**Published:** 2026-06-19

**Authors:** Zhangwei Lv, Haobo Zhang, Yinian Yang, Yuxian Wei, Xu Lei

**Affiliations:** 1 Sleep and NeuroImaging Center, Faculty of Psychology, Southwest University, Chongqing, China; 2 Key Laboratory of Cognition and Personality (Southwest University), Ministry of Education, Chongqing, China

**Keywords:** DTI-ALPS, glymphatic function, insomnia disorder, structural-functional coupling, subcortical network, working memory

## Abstract

**Background:**

Working memory (WM) deficits are frequently observed in patients with insomnia disorder (ID), but their neural basis is unclear. Glymphatic dysfunction and disrupted structural-functional coupling have been implicated, yet they have rarely been examined together, particularly in clinical populations.

**Methods:**

We conducted a multimodal MRI study in 391 ID patients. Glymphatic function was estimated using the diffusion tensor image analysis along the perivascular space (DTI-ALPS). The SFC was derived by correlating structural connectivity and functional connectivity. WM was measured by the longest span on the digit span backward task. Partial correlations and mediation analyses were performed to examine associations among sleep quality (Pittsburgh Sleep Quality Index, PSQI), DTI-ALPS, SFC, and WM performance.

**Results:**

DTI-ALPS was negatively correlated with PSQI (*r* = −0.17, *p* = 0.006), indicating reduced glymphatic clearance with poorer sleep quality. Global SFC was positively associated with DTI-ALPS (*r* = 0.32, pFDR < 0.001), but not with WM (*r* = 0.01, *p* = 0.84). At the network level, SFC within the subcortical network (Sub-SFC) correlated with both DTI-ALPS (*r* = 0.29, pFDR < 0.001) and WM performance (*r* = 0.28, pFDR < 0.001). Mediation analysis revealed that DTI-ALPS and Sub-SFC jointly mediated the association between PSQI and WM performance, with a significant indirect effect (indirect effect = −0.074).

**Conclusions:**

This study provides novel evidence that impaired glymphatic clearance and reduced Sub-SFC form key neural pathways linking poor sleep quality to working-memory deficits in ID, and that DTI-ALPS and Sub-SFC may serve as useful biomarkers of cognitive vulnerability.

## Introduction

Insomnia disorder (ID), characterized by persistent difficulties in initiating or maintaining sleep, is a prevalent condition affecting ~10% of the population worldwide (American Psychiatric Association, 2013; Morin et al., 2015). While traditionally viewed as a sleep-related problem, ID is increasingly recognized for its broader impact on daytime functioning (Zhang et al., 2024). Among its cognitive consequences, working memory (WM) impairment is particularly prominent, with patients frequently exhibiting reduced ability to maintain and manipulate information (Fortier-Brochu, Beaulieu-Bonneau, Ivers, & Morin, 2012). These deficits persist even in the absence of comorbid mood disorders and are associated with broad functional impairment in daily life (Lv, Zhang, Fan, Wei, et al., 2025). However, the neural mechanisms underlying WM deficits in ID remain poorly understood.

Mounting evidence suggests that WM performance depends not only on intact neural circuits but also on physiological systems that ensure neural efficiency and metabolic balance. Poor sleep can disrupt several of these systems, including neuroplasticity, synaptic regulation, and waste clearance, ultimately impairing cognitive function (Chen & Wilson, 2017). Among these, the glymphatic system has garnered particular interest as a sleep-dependent waste clearance pathway that removes neurotoxic byproducts, such as β-amyloid (Aβ), accumulated during wakefulness (Nir et al., 2017). Impairments in this system, caused by sleep deprivation or fragmentation, may reduce clearance efficiency and thereby contribute to WM deficits (Xie et al., 2013). As such, glymphatic dysfunction may serve as a key physiological mechanism linking insomnia to impaired cognitive performance.

The glymphatic system, a glia-mediated waste clearance pathway, facilitates the removal of metabolic waste from the central nervous system via cerebrospinal-interstitial fluid exchange (Iliff et al., 2012). Its activity is tightly coupled with the sleep–wake cycle (Xie et al., 2013). During slow-wave sleep, the system becomes highly active, with animal studies showing a 95% increase in clearance efficiency and a twofold increase in the removal of neurotoxic substances compared to wakefulness (Xie et al., 2013). This sleep-dependent process plays a vital role in maintaining brain homeostasis, particularly through the clearance of soluble pathological proteins such as Aβ and tau oligomers. Impaired glymphatic function may result in the accumulation of toxic proteins, including Aβ plaques, thereby contributing to cognitive decline and heightening the risk of neurodegenerative disorders (Hsu et al., 2023). Recent clinical evidence also indicates that sleep deprivation impairs glymphatic clearance, leading to abnormal accumulation of misfolded proteins in synaptic regions, a pathological hallmark associated with cognitive decline (Bishir et al., 2020). This highlights the need for noninvasive neuroimaging approaches to evaluate glymphatic function and its role in insomnia-related cognitive impairment.

Accurate evaluation of glymphatic function in the human brain poses notable methodological challenges. Traditional approaches such as intrathecal or intravenous administration of gadolinium-based contrast agents (GBCAs) have been used to trace perivascular clearance pathways, but their invasiveness limits their feasibility for large-scale or clinical application (Ringstad, Vatnehol, & Eide, 2017). To address this, Taoka et al. (2017) introduced a noninvasive alternative: the diffusion tensor image analysis along the perivascular space (DTI-ALPS), which quantifies water diffusivity along the perivascular spaces in the lateral ventricles (*x*-axis in the standard MNI space) using standard DTI sequences (Taoka et al., 2017). The DTI-ALPS indirectly reflects cerebrospinal fluid–interstitial fluid exchange and is increasingly recognized as a surrogate biomarker of glymphatic function. The DTI-ALPS has demonstrated strong construct validity, showing a robust negative correlation with glymphatic MRI based on intrathecal GBCAs (*r* = −0.82) (Zhang et al., 2021), and excellent test–retest reliability under standardized scanning conditions (ICC = 0.89–0.95) (Taoka et al., 2022). Leveraging these advantages, the DTI-ALPS has been applied in studies of Alzheimer’s disease and cerebral small vessel disease (Ota et al., 2022; Shen et al., 2022; Zhang et al., 2021). Notably, recent sleep research has revealed significant associations between the DTI-ALPS and sleep continuity, as well as its predictive value for cognitive outcomes such as memory performance (Clark et al., 2024). These findings support its use as a sensitive, noninvasive tool for assessing glymphatic function in relation to cognitive vulnerability in insomnia.

Beyond glymphatic dysfunction, neuroimaging studies have shown that ID is also linked to structural and functional alterations in the brain, particularly in regions underlying memory and executive control (Koo et al., 2017; Perrier, Chavoix, & Bocca, 2015; Yang et al., 2023; Zhang et al., 2024). For instance, disruptions in functional connectivity (FC) across frontal and temporal areas, as well as gray matter atrophy in the hippocampus and basal ganglia, have been associated with attentional and memory deficits (Lv et al., 2025; Perrier et al., 2015; Yang et al., 2023). However, most of these studies examined structural or functional changes in isolation, thereby neglecting their dynamic interaction (Park & Friston, 2013).

Structural-functional coupling (SFC) quantifies the degree to which functional communication between brain regions follows their anatomical wiring (Honey, Kötter, Breakspear, & Sporns, 2007). Recent research has shown that SFC provides stronger explanatory power than unimodal metrics in capturing cognitive variability (Liu et al., 2024; Wu et al., 2023) and detecting neural dysfunction in psychiatric populations (Zamani Esfahlani et al., 2022; Zhao et al., 2021). Importantly, SFC correlates with both sleep quality and memory performance (Ma et al., 2025), suggesting that insomnia may weaken the alignment between brain structure and function, thus contributing to cognitive decline. Since SFC varies across functional networks, a regionally-specific analysis may offer more insight than global indices – particularly for WM, which relies on coordinated activity between cortical and subcortical regions (Popp et al., 2024). Thus, a network-based analysis of SFC may offer more nuanced insights than global SFC measures, especially for cognitive domains such as WM that rely on dynamic coordination between cortical and subcortical networks (Fotiadis et al., 2024). Subcortical regions, such as the hippocampus, thalamus, and basal ganglia, are key hubs for memory, attention, and arousal regulation, all of which are frequently impaired in ID (Gent, Bandarabadi, Herrera, & Adamantidis, 2018; Rasch & Born, 2013). Disrupted SFC within these regions has been linked to poor sleep and WM dysfunction (Wang et al., 2024). Taken together, these findings highlight subcortical SFC as a promising neural biomarker linking sleep disruption to cognitive vulnerability in ID patients.

In summary, sleep disruption in insomnia disorder may compromise cognition not only through altered neural activity but also through physiological processes that sustain neural efficiency, including glymphatic waste clearance. At the same time, insomnia has been linked to altered coordination between brain structure and function, captured by SFC, a multimodal marker that relates to individual differences in memory performance. Yet, how glymphatic function and SFC are jointly implicated in working memory deficits in insomnia remains largely unknown.

Here, we combined DTI-ALPS as a noninvasive proxy of glymphatic clearance with multimodal connectomics to quantify SFC, focusing on subcortical circuits central to working memory. Conceptually, glymphatic clearance reflects an upstream homeostatic process that may shape network-level coupling by influencing white-matter–dependent communication pathways, and recent work has linked sleep quality, DTI-ALPS, and SFC within a unified framework (Ma et al., 2025). Building on this line of evidence, we tested whether sleep quality relates to working memory performance through DTI-ALPS and subcortical SFC. We expected poorer sleep quality to be associated with worse working memory performance, and lower DTI-ALPS and reduced SFC to be associated with both poorer sleep and poorer working memory.

## Patients and methods

### Study participants and procedures

This study recruited participants aged 18–75 years with ID from multiple hospitals in Chongqing, China, through both inpatient and outpatient departments, with the recruitment period lasting from March 2023 to January 2025. Eligible participants were screened by experienced psychiatrists through a semi-structured interview based on the diagnostic criteria for insomnia disorder outlined in the *Diagnostic and Statistical Manual of Mental Disorders, Fifth Edition (DSM-5)*. Inclusion criteria for participants with ID were (1) ID diagnosis according to DSM-5 criteria for ID, (2) absence of comorbid psychiatric or other sleep disorders, (3) be between 18 and 75 years old, (4) the sleep disturbance occurred at least 1 year, and (5) have no major physical illnesses or contraindications for MRI scanning. This study adhered to the ethical principles of the *Declaration of Helsinki*, and written informed consent was obtained from all participants. The study protocol was approved by the Human Research Ethics Committee of the Faculty of Psychology, Southwest University (Approval No.: H21070 and H24061).

**Figure 1.**
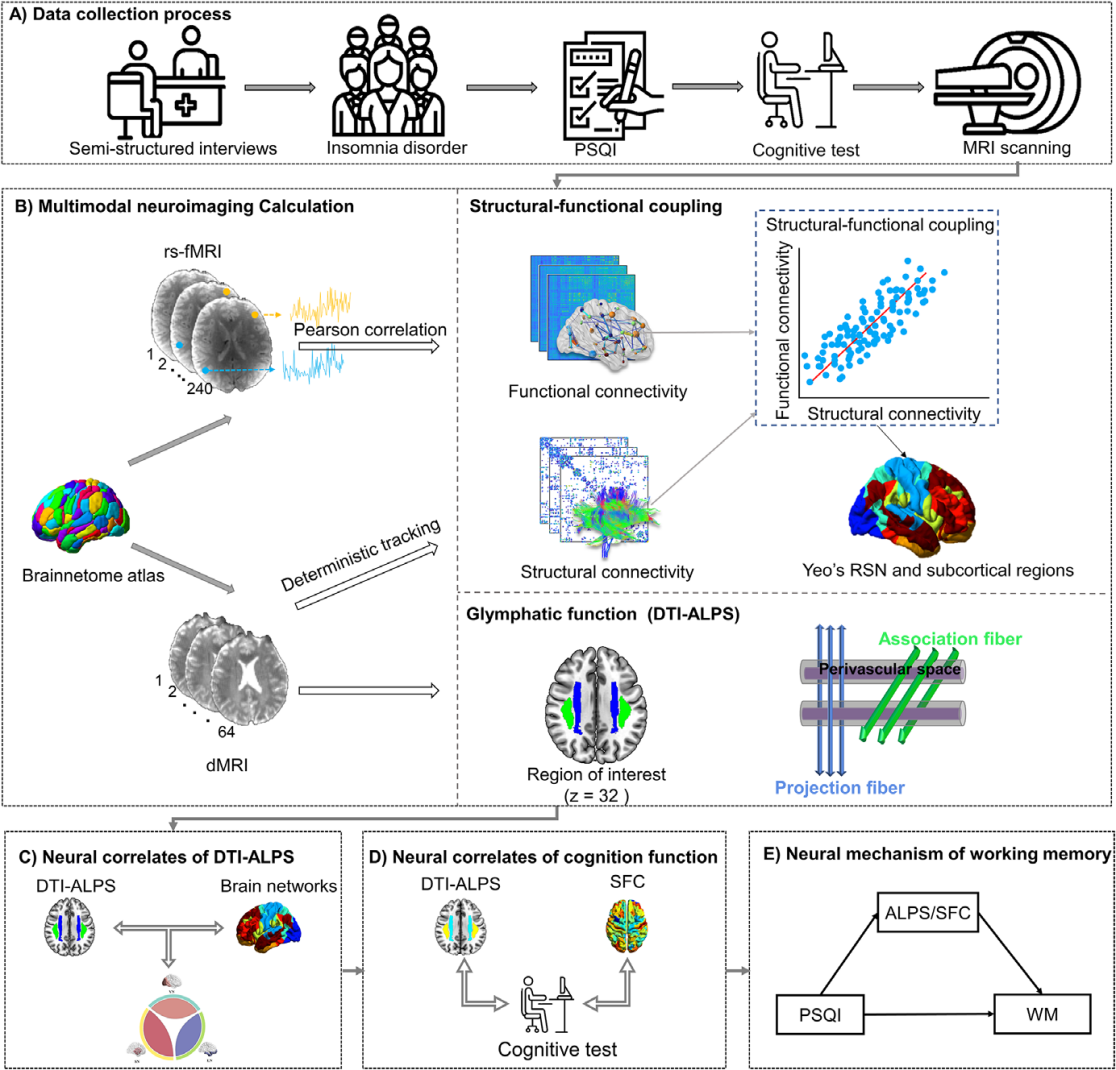
Study design and analysis procedure. (a) Conducting behavioral assessments and multimodal MRI scanning after the enrollment of ID patients. (b) Constructing structural and functional brain networks based on multimodal data. (c) Defining regions of interest in the dMRI using an atlas-based approach and calculating the DTI-ALPS. (d) Partial correlation analysis examining associations between brain network connectivity properties and DTI-ALPS. (e) Partial correlation analysis examining associations between SFC/DTI-ALPS and WM performance. (f) Integrated analysis of SFC and DTI-ALPS elucidating the neurocognitive mechanisms underlying working memory impairment in ID patients. Abbreviations: SFC: structural-functional coupling; DTI-ALPS: diffusion tensor image analysis along the perivascular space; PSQI: Pittsburgh Sleep Quality. Figure 1. long description.

After participant enrollment, cognitive assessments and multimodal MRI data collection were conducted. Diffusion MRI (dMRI) was used to calculate the DTI-ALPS as an indirect proxy of glymphatic function, while structural connectivity network (SCN) and functional connectivity network (FCN) were constructed from dMRI and resting-state fMRI (rs-fMRI), respectively. We first examined the association between the DTI-ALPS and sleep quality using partial correlation analysis. Next, we investigated how DTI-ALPS relates to brain network connectivity in ID patients. Finally, to explore whether glymphatic function and SFC jointly contribute to the neural mechanisms linking sleep quality and WM performance, we tested a mediation model with sleep quality as the independent variable, WM performance as the dependent variable, and SFC and/or the DTI-ALPS as potential mediators. The overall workflow and analysis procedure are illustrated in Figure 1.

**Table 1.** Demographic characteristics and sleep quality measures *M* (SD) of patients with ID included in the final analysis (*N* = 391) Table 1. long description.

Characteristic	Number	*M* (SD) / *n* (%)	Minimum	Maximum
Demographic information				
Age (years)	391	43.15 ± 15.39	18.42	75.33
Gender, women, *n* (%)	391	224 (57.29)		
Handedness, left-handed, *n* (%)	391	8 (2.04)		
Pittsburgh Sleep Quality Index				
Total scores	265	11.63 ± 3.47	5	21
Sleep quality	265	2.48 ± 0.64	1	3
Sleep latency	265	2.11 ± 0.86	0	3
Sleep duration	265	1.21 ± 1.18	0	3
Sleep efficiency	265	1.10 ± 1.12	0	3
Sleep disturbances	265	1.49 ± 0.60	0	3
Sleep medication	265	0.82 ± 1.22	0	3
Daytime dysfunction	265	2.42 ± 0.81	0	3
Subjective sleep indicators				
Total sleep time (hours)	332	7.11 ± 1.72	1.11	16.85
Sleep onset latency (hours)	332	0.40 ± 0.49	0.01	4.20
Sleep efficiency (%)	332	92.57 ± 7.86	31.90	99.94

### Behavioral assessment

The Pittsburgh Sleep Quality Index (PSQI) was used to assess sleep quality and related problems over the past month. It covers seven components: (1) sleep quality, (2) sleep latency, (3) sleep duration, (4) sleep efficiency, (5) sleep disturbance, (6) use of sleep medication, and (7) daytime dysfunction (Buysse et al., 1989). The PSQI is commonly utilized to assess sleep quality in the general population and in patients with sleep-related or psychiatric conditions. It includes items rated from 0 to 3, producing a total score between 0 and 21.

WM performance was assessed using the digit span backward task, a widely used WM measure and a core subtest in both the Wechsler Memory Scale and the Wechsler Adult Intelligence Scale. Participants were required to recall sequences of digits in reverse order, with sequence length gradually increasing. WM performance was quantified using the two-error maximum length (TEML), defined as the longest sequence correctly recalled before two consecutive errors occurred at the same length (Woods et al., 2011). This experimental paradigm was adapted from existing studies and implemented using the jsPsych platform (https://www.jspsych.org).

### Multimodal neuroimaging data acquisition and preprocessing

The MRI data in this study were acquired from two sites. The Southwest University site (SWU dataset) was acquired at Southwest University using a 3T Siemens Prisma-fit scanner (Siemens, Erlangen, Germany), whereas the Chongqing United Medical Imaging site (UMI dataset) was obtained at Chongqing United Medical Imaging using a 3.0T GE SIGNA Pioneer MRI scanner. Both datasets included T1-weighted (T1w) structural images, resting-state fMRI (rs-fMRI), and dMRI data. Detailed scanning parameters are provided in Supplementary Table S1.

In this study, rs-fMRI data processing was performed using the Data Processing and Analysis of Brain Imaging (DPABI V8.2, http://rfmri.org/DPABI) toolbox (Yan, Wang, Zuo, & Zang, 2016). dMRI preprocessing was conducted using the FMRIB Software Library (FSL 5.0, https://fsl.fmrib.ox.ac.uk/fsl/docs/#/) following the standard pipeline of the Pipeline for Analyzing braiN Diffusion Images (PANDA) toolbox (Cui et al., 2013). A detailed description of the rs-fMRI and dMRI preprocessing steps is provided in the Supplementary Materials.

### Construction of the whole-brain network

#### Functional connectivity network construction

To construct the FCN, we first mapped each participant’s preprocessed rs-fMRI data onto the Brainnetome atlas (Fan et al., 2016), which parcellates the gray matter into 246 regions (210 cortical and 36 subcortical). The atlas provides information on their overlap with the Yeo resting-state networks (RSNs) (Yeo et al., 2011), which comprise seven cortical networks: the visual network (VN), somatomotor network (SMN), dorsal attention network (DAN), ventral attention network (VAN), limbic network (LN), frontoparietal network (FPN), and default mode network (DMN). Since the original Yeo RSNs do not include subcortical structures, we classified the 36 subcortical regions in the Brainnetome atlas collectively as a separate subcortical network (Sub) to ensure comprehensive coverage of the whole brain. Detailed information on the Brainnetome atlas parcellation and network affiliation is provided in Supplementary Table S2.

Next, we averaged the BOLD time series across all voxels within each brain region based on the Brainnetome atlas. The FC between two regions (i.e., the network edges) was defined as the Pearson correlation coefficient between their mean BOLD time series. To enhance the normality of the data, we applied the Fisher’s z transformation to the correlation coefficients and used the transformed values as edge weights. This process resulted in the construction of a 246 × 246 FCN for each participant (Figure 1b).

#### Structural connectivity network construction

The SCN adopted the same brain region definitions as the FCN. Specifically, we first registered the preprocessed dMRI data to the standard MNI space and applied deterministic fiber tracking using the Fiber Assignment by Continuous Tracking (FACT) algorithm within the white matter mask. To minimize false-positive connections at the individual level, fiber tracking was stopped when the angle between two consecutive directions exceeded 45° or when the fractional anisotropy (FA) dropped below 0.2. Next, we inverse-transformed the Brainnetome atlas from MNI space to the individual diffusion space, where the parcellated brain regions defined by the atlas served as network nodes, generating an individual-space parcellation image for each participant. To define network edges between the 246 brain regions in the Brainnetome atlas, we considered a connection to exist if any streamline passed through or terminated in both regions. Additionally, a structural connection was only considered valid if at least three streamlines with both endpoints in the respective brain regions were detected. Finally, by computing the number of streamlines between each node pair, we constructed a 246 × 246 SCN for each participant (Figure 1b).

#### Coupling between functional and structural brain networks

At the global level, we extracted all valid SC (≥3 streamlines) from the individual SCN, forming a one-dimensional vector, and obtained the corresponding FC from the FCN. The SFC strength was then computed as the Spearman correlation coefficient between these two vectors. At the network level, we computed within-network SFC based on predefined RSNs. Specifically, for each network, we calculated the Spearman correlation between valid SC and corresponding FC values for all pairs of regions within that network, thereby capturing network-specific SFC.

### Calculation of the DTI-ALPS

**Figure 2.**
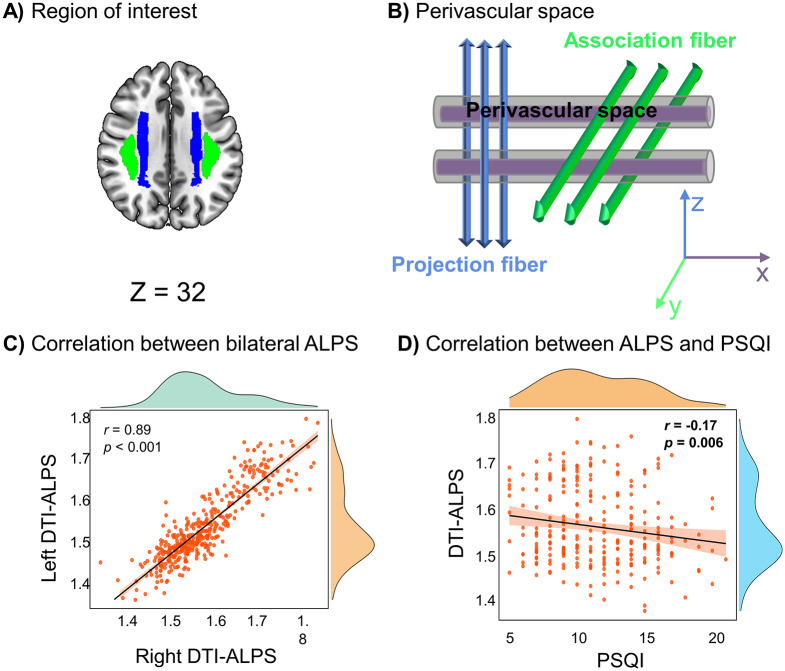
Estimation of DTI-ALPS and its correlation with sleep quality. (a) Brain regions analyzed from the ICBM-DTI-81 atlas, specifically in the superior/posterior corona radiata (blue) and superior longitudinal fasciculus (green, *Z* = 32 mm). (b) Schematic representation of the perivascular space, depicting the association and projection fibers involved in DTI-ALPS calculations. (c) Positive correlation between the left and right DTI-ALPS indices. (d) Negative correlation between DTI-ALPS and PSQI. Figure 2. long description.

To assess glymphatic clearance function in the brain, we adopted a method proposed by Taoka et al. (2017) based on dMRI (Taoka et al., 2017). This technique evaluates anisotropic water diffusion along perivascular pathways and derives a proxy of glymphatic function known as the DTI-ALPS. Although the DTI-ALPS is recommended for clinical assessment of glymphatic clearance function, its results are sensitive to head motion during scanning and dependent on the choice of *b*-values (Taoka et al., 2017). To address these challenges and enhance data reliability, we selected a *b*-value of 1000 s/mm^2^ and implemented strict head motion criteria. Specifically, during preprocessing, subjects with head motion exceeding 2 mm in displacement, 2° in rotation, or a mean frame-wise displacement (FD) larger than 0.25 mm were excluded from subsequent analyses. We selected a *b*-value of 1000 s/mm^2^ based on methodological considerations. First, lower *b*-values offer a higher signal-to-noise ratio and greater sensitivity to relatively fast water diffusion, such as that occurring along perivascular pathways. Second, prior studies have reported more stable and interpretable DTI-ALPS measurements at *b* = 1000 s/mm^2^, particularly in clinical populations (Taoka et al., 2017). Furthermore, to minimize subjective bias from manual region of interest (ROI) delineation in DTI-ALPS calculation, we employed an automated atlas-based approach (Hsu et al., 2023; Ma et al., 2025; Yokota et al., 2019). The analytical pipeline consisted of: (1) co-registering FA and directional diffusivity maps (*x*-, *y*-, and *z*-axes) from native space to the standard MNI space; (2) extracting predefined ROIs from the ICBM-DTI-81 atlas in MNI space, specifically targeting projection and association fibers adjacent to the lateral ventricles; (3) and applying a threshold mask (FA > 0.2) within each ROI to exclude voxels potentially contaminated by cerebrospinal fluid (http://bmap.ucla.edu/portfolio/atlases/ICBM_DTI-81_Atlas/). Projection and association fiber ROIs were defined at the superior/posterior corona radiata and superior longitudinal fasciculus, respectively (Figure 2a). The DTI-ALPS was computed as the mean of *x*-axis diffusivity in the projection fiber area (D*_xproj_*) and *x*-axis diffusivity in the association fiber area (D*_xassoc_*), divided by the mean of *y*-axis diffusivity in the projection fiber area (D*_yproj_*) and *z*-axis diffusivity in the association fiber area (D*_zassoc_*).

$$\mathrm{DTI}-\mathrm{DTI}-\mathrm{ALPS}=\frac{\mathrm{mean}\left(D_{xproj};D_{xassoc}\right)}{\mathrm{mean}\left(D_{yproj};D_{zassoc}\right)}$$

To assess hemispheric effects, we separately calculated DTI-ALPS indices for left and right ROIs. Then these indices were averaged to obtain the bilateral index for subsequent analyses.

### Statistical analyses

All data analyses were conducted using MATLAB 2022b and SPSS 26. Because the imaging data were collected from two scanning sites (SWU and UMI), we applied ComBat harmonization (https://github.com/Jfortin1/ComBatHarmonization) to the SC, FC, and SFC matrices to reduce site-related variability (Fortin et al., 2018; Lv, Zhang, Fan, Chen, et al., 2025). We first performed partial correlation analyses (controlling for age, gender, and scanning site) to assess the relationship between sleep quality (PSQI) and DTI-ALPS. Next, we examined the association between DTI-ALPS and SFC at both the global and network levels. We then assessed how DTI-ALPS and SFC related to WM performance. Multiple comparisons were corrected using the false discovery rate (FDR) method.

Based on these significant associations, we conducted mediation analyses using the PROCESS macro (Model 4) in SPSS to test DTI-ALPS or SFC as mediators between PSQI and WM. Given that the DTI-ALPS reflects glymphatic clearance function, a physiological process facilitating metabolic waste removal from the brain, and that glymphatic function may modulate brain-wide network connectivity (Fultz et al., 2019; Ma et al., 2025), we hypothesized a directional pathway in which DTI-ALPS influences SFC. Finally, we constructed a serial mediation model (Model 6) to examine a directional pathway in which PSQI influenced WM through both DTI-ALPS and SFC. The indirect effects were evaluated using bias-corrected bootstrapping (5,000 resamples). Fisher’s *r*-to-*z* transformation was applied to SFC values before mediation and moderation analyses to better approximate normality.

## Results

### Demographic and clinical characteristics

A total of 431 patients diagnosed with ID were initially recruited for MRI scanning. After excluding 18 participants due to corrupted or missing MRI data and 22 for excessive head motion, 391 participants were retained for analysis (mean age = 43.15, SD = 15.39; 57.29% female, *n* = 224). A total of 265 participants completed the PSQI (nonresponders were primarily older adults with comprehension difficulties), with 96.98% scoring >5, indicating widespread clinically significant sleep disturbances. A detailed enrollment and exclusion flow, including the number of participants excluded at each step and the reasons for exclusion, has been added to Figure S1.

### Association between DTI-ALPS and sleep parameters

Before reporting the DTI-ALPS findings, we first evaluated and corrected site effects. ComBat harmonization substantially reduced site-related variance in FC (*t* = 114.90, *p* < 0.001), with smaller nonsignificant reductions for SC and SFC (Table S3 and Figure S2). For DTI-ALPS, which is a low-dimensional metric, regressing out site, age, and gender effectively removed site differences (Figure S2d). This study found a significant correlation between the left and right DTI-ALPS indices (*r* = 0.89, *p* < 0.001, Figure 2c), indicating high consistency across hemispheres. After controlling for gender, age, and site, partial correlation analysis revealed a significant negative correlation between the DTI-ALPS and the PSQI total score (*r* = −0.17, *p* = 0.006, Figure 2d). Further analysis of the PSQI subdomains showed significant negative correlations between the DTI-ALPS and sleep efficiency (*r* = −0.17, *pFDR* = 0.02), as well as the use of sleep medications (*r* = −0.17, *pFDR* = 0.02, Figure S3).

### Relationship between DTI-ALPS and brain network connectivity

**Figure 3.**
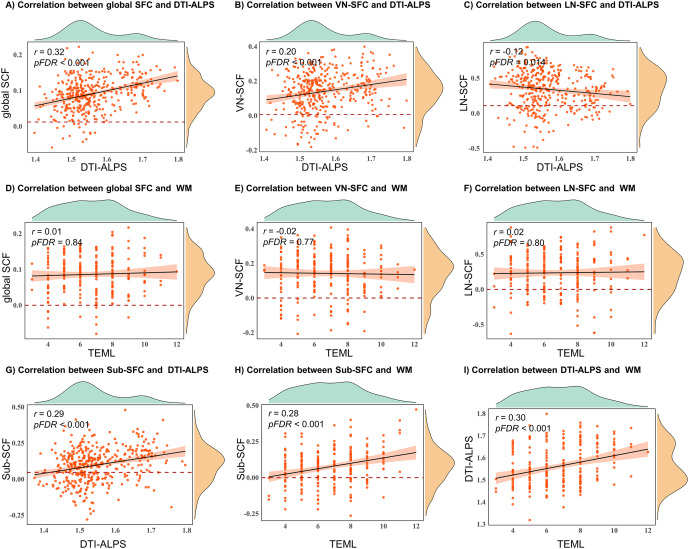
Relationship between SFC, DTI-ALPS, and WM performance. (a–c) Correlation between global SFC, VN-SFC, and LN-SFC and DTI-ALPS. (d–f) Correlation between global SFC, VN-SFC, and LN-SFC and WM performance. (g) Correlation between Sub-SFC and the DTI-ALPS. (h) Correlation between Sub-SFC and WM performance. (i) Correlation between the DTI-ALPS and WM performance. Multiple comparisons were corrected using the FDR correction. Abbreviations: SFC: structural-functional coupling; DTI-ALPS: diffusion tensor image analysis along the perivascular space; VN: visual network; WM: working memory; LN: limbic network; TEML: two-error maximum length in working memory task; Sub: subcortical network. Figure 3. long description.

At both the global and network levels, partial correlations between SFC and DTI-ALPS were computed while controlling for gender, age, and scanning site. The results revealed significant positive correlations between DTI-ALPS and global SFC (*r* = 0.32, pFDR < 0.001; Figure 3a), SFC within VN (VN-SFC; *r* = 0.20, pFDR < 0.001; Figure 3b), and SFC within Sub (Sub-SFC; *r* = 0.29, pFDR < 0.001; Figure 3g). In contrast, a significant negative correlation was observed with the SFC within LN (LN-SFC; *r* = −0.12, pFDR = 0.014; Figure 3c). Considering the possibility of hemispheric asymmetry, we further examined the associations between left and right DTI-ALPS and network-level SFC. The findings were highly consistent with those obtained using mean DTI-ALPS (left: *r* = 0.31, pFDR < 0.001; right: *r* = 0.28, pFDR < 0.001; Figure S4).

### Relationship between SFC/DTI-ALPS and cognitive function

Among the 216 ID patients who completed cognitive assessments, we investigated the associations between SFC and WM performance using partial correlation analyses. The results revealed that only Sub-SFC showed a significant positive correlation with WM performance (*r* = 0.28, pFDR < 0.001; Figure 3h), while no significant partial correlations were observed for other measures (Figure 3d–f). Using the same partial correlation analysis, the DTI-ALPS was also found to be significantly associated with WM performance (*r* = 0.30, pFDR < 0.001; Figure 3i), supporting the association between better WM and stronger glymphatic clearance. This pattern was fully preserved when analyzing the two hemispheres separately, with significant associations observed for both the left (*r* = 0.33, pFDR < 0.001) and right (*r* = 0.26, pFDR < 0.001) DTI-ALPS indices (Figure S4). Based on these findings, no significant association was observed between global SFC and WM performance. To further explore the neural mechanisms underlying the relationship between sleep quality and WM performance in ID patients, subsequent mediation analyses in this study focused on examining the mediating roles of Sub-SFC and the DTI-ALPS in the association between sleep quality and WM performance in individuals with ID.

### Network-specific effects of SFC on working memory

**Figure 4.**
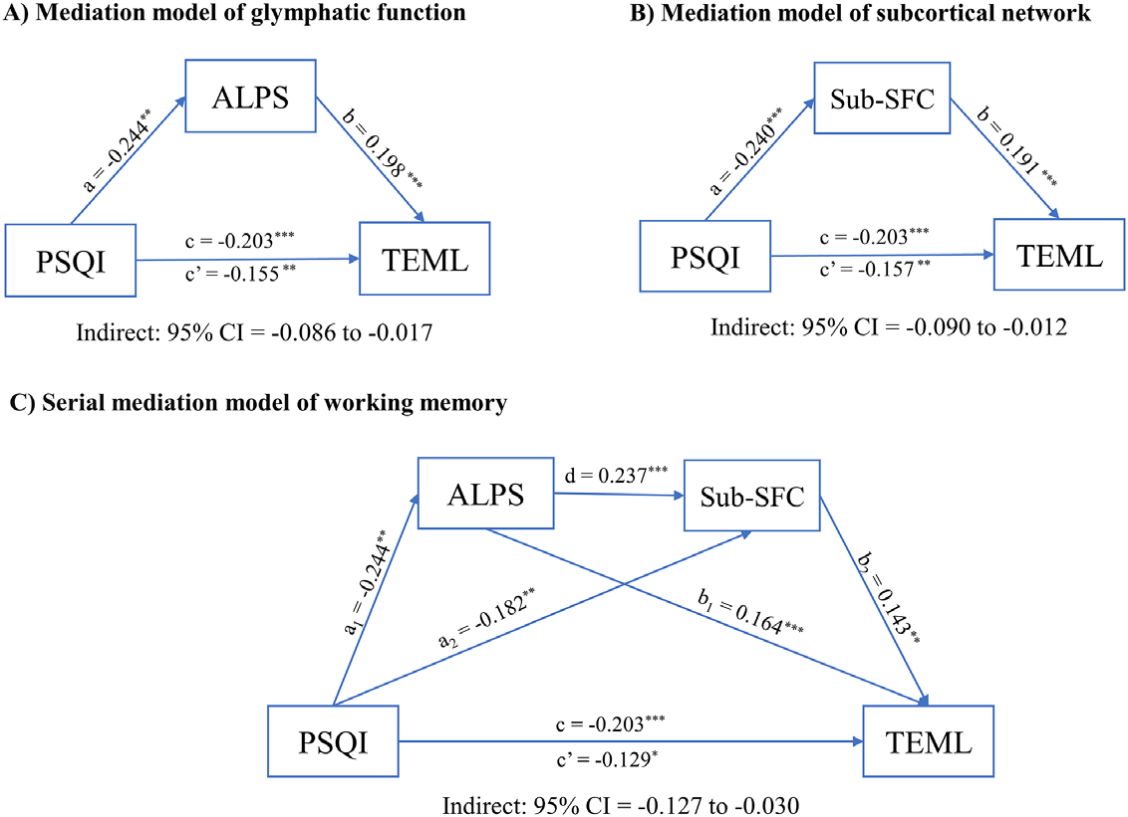
Mediation of working memory. (a) DTI-ALPS significantly mediated the relationship between PSQI and TEML. (b) Sub-SFC significantly mediated the relationship between PSQI and TEML. (c) The DTI-ALPS and Sub-SFC together mediated the relationship between PSQI and TEML. ***p < 0.001, **p < 0.01, *p < 0.05. Abbreviations: PSQI: Pittsburgh Sleep Quality Index; SFC: structural-functional coupling; DTI-ALPS: diffusion tensor image analysis along the perivascular space; TEML: two-error maximum length in working memory task; Sub: subcortical network. Figure 4. long description.

We first established a mediation model with PSQI as the independent variable, TEML as the dependent variable, and the DTI-ALPS as the mediator. The results showed that the DTI-ALPS significantly negatively mediated the relationship between PSQI and TEML (*β* = −0.048 [23.65% of the total effect], SE = 0.018, bootstrapped 95% CI = −0.086 to −0.017, Figure 4a). Next, we modified the mediation model by changing the mediator to the Sub-SFC while keeping PSQI as the independent variable and TEML as the dependent variable. The results showed that Sub-SFC significantly negatively mediated the relationship between PSQI and TEML (*β* = −0.051 [25.12% of the total effect], SE = 0.020, bootstrapped 95% CI = −0.090 to −0.012, Figure 4b). These findings indicate that poorer sleep quality was associated with worse WM performance in ID patients, with Sub-SFC partially mediating this association. To assess whether the moderation pattern reported in healthy older adults (Ma et al., 2025) also appears in our clinical insomnia sample, we conducted an exploratory moderated mediation analysis. Participants were divided into lower- and higher-PSQI subgroups based on a median split, indexing relatively less versus more severe sleep disturbance. In PROCESS Model 14, DTI-ALPS served as the predictor, Sub-SFC as the mediator, TEML as the outcome, and the PSQI subgroup as a moderator of the association between Sub-SFC and WM. The interaction showed a trend-level effect (*β* = −0.225, *p* = 0.076; index of moderated mediation = −0.058, bootstrapped 95% CI =[−0.140, 0.009]; Figure S5a), suggesting potential variation in the coupling–memory relationship across PSQI levels. Follow-up mediation analyses further indicated that the indirect effect via Sub-SFC emerged only in the lower-PSQI subgroup (Figure S5b–c).

Finally, to investigate the neural mechanisms underlying WM impairment through the combined effects of network-specific SFC and DTI-ALPS, we established a serial mediation model with sleep quality as the independent variable, WM performance as the dependent variable, and DTI-ALPS and Sub-SFC as mediators. The results revealed a significant direct effect of PSQI on TEML, indicating that poorer sleep quality is associated with worse WM performance (*β* = −0.129 (63.55% of the total effect), SE = 0.053, 95% CI = −0.233 to −0.025). Importantly, the relationship between PSQI and TEML was significantly mediated by both DTI-ALPS and Sub-SFC (*β* = −0.074 [36.62% of the total effect], SE = 0.025, bootstrapped 95% CI = −0.127 to −0.030, Figure 4c). Specifically, the indirect effect via DTI-ALPS was significant (*β* = −0.040 [19.71% of the total effect], SE = 0.015, bootstrapped 95% CI = −0.073 to −0.012), while the indirect effect via Sub-SFC was also significant (*β* = −0.026 [12.44% of the total effect], SE = 0.026, bootstrapped 95% CI = −0.061 to −0.001). Furthermore, a serial mediation path was identified, where PSQI influenced TEML through both DTI-ALPS and Sub-SFC (*β* = −0.008 [4.09% of the total effect], SE = 0.006, bootstrapped 95% CI = −0.023 to −0.001). Among the three models, the single-mediator model involving Sub-SFC (Model 2) yielded the lowest AIC and BIC (AIC = 994.41, BIC = 1017.50), and was therefore considered the best-fitting model according to information criteria (Table S4). We additionally confirmed that the mediation effects remained robust when left and right DTI-ALPS values were analyzed separately, yielding patterns highly consistent with the results based on mean DTI-ALPS (Figure S6). Given prior evidence that handedness can influence hemispheric structural and functional asymmetries (Tejavibulya et al., 2025), we repeated both the partial correlation analyses and the serial mediation model while controlling for handedness. When handedness was included as a covariate, the partial correlations among the main variables remained highly consistent with the original results. Similarly, adding handedness to the serial mediation model produced an indirect effect that was virtually identical to the primary analysis (*β* = −0.075, 36.94% of the total effect, SE = 0.025, bootstrapped 95% CI = −0.127 to −0.030). These findings suggest that the influence of sleep quality on WM performance is partially transmitted via alterations in DTI-ALPS and Sub-SFC, which serve as key neurophysiological mediators.

## Discussion

This study systematically investigated the complex relationships between sleep quality, DTI-ALPS, and SFC, as well as their impact on WM in ID patients, based on multimodal neuroimaging data. First, we found that poor sleep quality in ID patients was significantly associated with reduced DTI-ALPS and SFC. Second, DTI-ALPS showed specific associations with multimodal brain network features, particularly stronger SFC within the visual network and subcortical network, which were linked to higher DTI-ALPS, while the opposite pattern was observed in the limbic network. Most importantly, both DTI-ALPS and SFC within the subcortical network were positively associated with WM performance and jointly mediated the relationship between sleep quality and WM. To our knowledge, this is the first clinical study to apply DTI-ALPS and SFC analysis in ID patients, providing novel evidence for the neurobiological mechanisms underlying WM impairment in this population.

### Sleep quality and DTI-ALPS

There is a close physiological relationship between sleep and the brain’s glymphatic function. Recent studies have found that the activity of the glymphatic system exhibits significant circadian rhythmicity, with a substantial increase during sleep and a marked suppression during wakefulness (Voumvourakis et al., 2023). In this study, we observed that poorer sleep quality was associated with reduced glymphatic function, a phenomenon that has been validated in multiple independent studies (Ma et al., 2025; Taoka et al., 2024). This result suggests that a decline in sleep quality may interfere with fluid convection in perivascular spaces, thereby affecting the efficiency of metabolic waste clearance (Carley & Farabi, 2016). The neuroprotective role of sleep largely depends on glymphatic clearance of neurotoxic substances, such as Aβ (Reddy & van der Werf, 2020). This study used the DTI-ALPS as an imaging marker for evaluating glymphatic function. This technique offers the advantage of being completely noninvasive and has shown good consistency with results obtained from traditional intrathecal contrast agent injection methods (Zhang et al., 2021). Notably, the high correlation between bilateral DTI-ALPS indices indicates robust spatial consistency and minimal hemispheric asymmetry in glymphatic measurements. This finding aligns with previous studies reporting comparable left–right consistency in both healthy individuals and patients with insomnia disorder (Xiong et al., 2025), supporting the reliability of the DTI-ALPS method. In addition, the overall DTI-ALPS values in our sample were within the expected range reported in prior literature, further validating the robustness of our measurement approach (Ma et al., 2025; Xiong et al., 2025). At the same time, the cross-sectional nature of our study does not allow us to determine the directionality of these associations. A bidirectional relationship between sleep and glymphatic function is also plausible (Hauglund & Nedergaard, 2025; Xiao, Marshall, & Yin, 2025). Chronic dysregulation of glymphatic clearance could promote the accumulation of neurotoxic metabolites, neuroinflammation, and vascular stiffness, which may in turn fragment sleep, alter sleep architecture, or increase nocturnal arousals (Cai et al., 2024). From this perspective, reduced glymphatic efficiency might not only be a consequence of poor sleep but could also contribute to the maintenance or worsening of sleep disturbances. Longitudinal and interventional studies that track changes in both sleep and DTI-ALPS over time will be needed to disentangle these potential causal pathways (Gottesman et al., 2024).

PSQI subdimension analyses showed that both decreased sleep efficiency and increased frequency of hypnotic medication use were significantly associated with reduced glymphatic function. This finding may be because low sleep efficiency is often accompanied by prominent features of sleep fragmentation (Dashti et al., 2016), including prolonged wake time after sleep onset and increased micro-arousal frequency. Existing research has shown that such fragmented sleep can significantly suppress slow-wave activity during non-REM sleep (Ferri et al., 2010). Given that the glymphatic system’s metabolic clearance function relies heavily on the rhythmic expansion of perivascular spaces driven by neurovascular coupling during slow-wave sleep, reduced slow-wave activity caused by sleep fragmentation directly hinders the cerebrospinal–interstitial fluid exchange process (Fultz et al., 2019). Additionally, although frequent use of sedative medications may improve difficulties in initiating sleep, it may also interfere with glymphatic clearance by altering sleep architecture (Antila et al., 2024). However, it should be noted that in the present study sleep quality was assessed solely through subjective reports. Subjective ratings of sleep efficiency and medication use can be affected by recall bias and insomnia-related sleep misperception (Lv, Zhang, Fan, Chen, et al., 2025), and therefore, our findings should be interpreted as reflecting associations between perceived sleep quality and glymphatic function rather than objectively quantified sleep architecture. Future multimodal studies integrating questionnaires with actigraphy and/or polysomnography are needed to determine whether similar glymphatic alterations emerge when sleep is characterized using objective parameters, such as total sleep time, slow-wave activity, or arousal indices.

### The role of SFC in supporting glymphatic function

At the global level, we observed a significant positive correlation between SFC and the DTI-ALPS, suggesting that stronger structural-functional coupling is associated with better glymphatic function. The SFC reflects the degree to which anatomical connections support functional communication, and may be particularly sensitive to the efficiency and adaptability of inter-regional information transfer. This integrative property likely contributes to SFC’s predictive advantage in cognitive performance and its sensitivity to pathological disruptions in brain networks (Liu et al., 2024; Wu et al., 2023).

At the network level, the association between SFC and glymphatic function showed distinct patterns across RSNs. Notably, VN-SFC and Sub-SFC were positively correlated with the DTI-ALPS, whereas LN-SFC was negatively correlated. These findings indicate that the relationship between structure–function alignment and glymphatic efficiency is network-specific and may vary along the brain’s functional hierarchy. This pattern aligns with the concept of a unimodal-to-transmodal gradient of structure–function decoupling (Facca, Del Felice, & Bertoldo, 2024; Valk et al., 2022). In unimodal systems such as the VN and somatosensory-related networks, higher SFC may support more stable neurovascular dynamics and facilitate glymphatic clearance, which could help maintain glymphatic efficiency even in the context of poor sleep. In contrast, elevated SFC within transmodal networks such as the LN may reflect a more rigid or maladaptive coupling pattern, potentially associated with reduced network flexibility and impaired glymphatic function (Deco et al., 2021). In patients with ID, such a network-specific imbalance may have particular relevance for pathophysiology (Fasiello et al., 2022). Stronger coupling in limbic circuits that are involved in emotional salience, arousal regulation, and rumination could contribute to persistent hyperarousal and difficulties disengaging from internally focused or affectively laden processing during the night, which in turn may be linked to both reduced glymphatic efficiency and poorer WM performance (Baglioni et al., 2014; Riemann et al., 2010). Thus, our findings suggest that altered SFC patterns in insomnia may reflect an insomnia-specific reconfiguration of SFC that affects both nocturnal clearance processes and daytime cognition.

### WM performance correlates of SFC and glymphatic function

Our results demonstrated a positive correlation between the DTI-ALPS and WM performance. A growing body of evidence indicates that maintaining glymphatic clearance of neurotoxic waste (e.g. Aβ) is essential for preserving cognitive function and promoting brain health, with memory performance appearing especially sensitive to such effects (Shen et al., 2022). Impairments in glymphatic function can lead to abnormal accumulation of Aβ, which has been identified as a central mechanism underlying memory decline (Voumvourakis et al., 2023). Notably, studies in animal models have demonstrated that enhancing glymphatic outflow reduces Aβ and tau deposition and improves memory performance in Alzheimer’s disease models (Lee et al., 2020). Collectively, these findings highlight the clinical potential of targeting glymphatic function as an intervention strategy to improve memory and possibly prevent dementia progression in ID patients.

At the global level, no significant association was found between SFC and WM performance. Although previous studies have reported associations between SFC and cognition, these findings are primarily based on nonclinical populations (Medaglia et al., 2018; Siow et al., 2022), limiting their generalizability. For instance, one study involving healthy older adults showed that sleep quality, as measured by the PSQI, moderated the relationship between global SFC and memory performance: the association was significant only among individuals with good sleep, and not among those with poor sleep quality (Ma et al., 2025). Our findings in a clinical insomnia sample are consistent with this perspective, showing that global SFC does not significantly predict WM performance under impaired sleep conditions. Moreover, the literature remains inconclusive regarding the relationship between global SFC and cognitive function. While some studies report that increased global SFC is associated with cognitive decline (Wang et al., 2018), others have suggested that it may support cognitive flexibility (Medaglia et al., 2018) and facilitate complex task execution (Griffa et al., 2022). These inconsistencies point to the limitations of using whole-brain average SFC measures, which may obscure critical regional variations in SFC. In contrast, network-specific SFC indices may provide more sensitive and informative markers of the neural basis of cognitive processing and offer better predictive utility for individual cognitive performance (Baum et al., 2020; Popp et al., 2024). Supporting this hypothesis, our further analysis revealed a significant positive association between Sub-SFC and WM performance. This finding underscores the importance of considering network-specific coupling metrics, particularly under pathological conditions such as ID, where the brain’s network dynamics may become more spatially localized and functionally specialized.

### Potential mechanisms underlying the impact of sleep quality on working memory performance

The main finding of this study is that glymphatic function and the Sub-SFC jointly mediate the relationship between sleep quality and WM performance in ID patients. Working memory, one of the core cognitive functions, refers to the ability to temporarily store and manipulate task-relevant information. It plays a crucial role in executive functions and emotional regulation, thereby setting fundamental limits on an individual’s cognitive abilities (Miller, Lundqvist, & Bastos, 2018). A wealth of research has shown that sleep deprivation negatively affects WM performance (Almarzouki et al., 2022; Wardle-Pinkston, Slavish, & Taylor, 2019; Xie et al., 2019). From a cognitive process perspective, several hypotheses have been proposed to explain this relationship, such as sleep deprivation potentially increasing retroactive interference or diverting attention, which in turn impairs memory capacity (Rasch & Born, 2013). However, there is still no consensus on the neural mechanisms underlying how sleep quality impacts WM. This study provides new insights through multimodal imaging data.

As previously mentioned, poor sleep quality typically leads to a reduction in extracellular volume, which decreases the rate of molecular transport (L. Xie et al., 2013), thereby impairing glymphatic system function. This impairment in the glymphatic system’s clearance ability results in the accumulation of toxic proteins (e.g. Aβ) and misfolded proteins, a process considered to be one of the core neural mechanisms underlying memory impairment (Voumvourakis et al., 2023). Therefore, our first mediation model suggests that poorer sleep quality was associated with reduced glymphatic function, which in turn was associated with worse WM performance.

The second mediation model suggests that Sub-SFC partially mediated the association between sleep quality and WM performance. According to the Brainnetome Atlas, subcortical regions, including the hippocampus, thalamus, basal ganglia, amygdala, cingulate gyrus, and insula, are responsible for coordinating perception, memory, emotion, motor control, and motivation. These regions are crucial for WM, particularly in maintaining information, directing attention, and filtering irrelevant stimuli (Fan et al., 2016; Sander, Lindenberger, & Werkle-Bergner, 2012). Previous studies have shown that regional SFC within subcortical regions (e.g. thalamus) is negatively correlated with insomnia severity in female patients (Wu et al., 2023), supporting our finding that poorer sleep quality is associated with lower Sub-SFC. Subcortical regions are typically part of unimodal networks and exhibit lower SFC than multimodal networks (e.g. DMN; Valk et al., 2022). However, for unimodal regions, tight structural-functional alignment is vital for efficient neural processing (Taoka et al., 2017; Zamani Esfahlani et al., 2022). High SFC enhances the capacity of structural pathways to support local functional demands, thereby improving the speed and fidelity of information transfer (Gu, Jamison, Sabuncu, & Kuceyeski, 2021). Thus, a stronger Sub-SFC may provide greater structural support and metabolic efficiency, enabling subcortical regions to execute WM-related tasks more effectively. In this way, sleep quality indirectly influences WM performance through its modulation of Sub-SFC. Poor sleep disrupts this coupling, thereby impairing the execution of information maintenance, attentional control, and emotional regulation within these regions.

Finally, this study identified that both glymphatic function and Sub-SFC jointly mediate the relationship between sleep quality and WM performance, with a positive association observed between glymphatic efficiency and Sub-SFC. Previous research has shown that glymphatic dysfunction impairs CSF clearance along perivascular pathways by narrowing perivascular spaces and reducing aquaporin-4 activity, which in turn hinders the removal of metabolic waste and contributes to white matter injury, including demyelination (Iliff et al., 2012). Such impairment may also lead to the accumulation of neurotoxic proteins, triggering neuroinflammatory or neurodegenerative processes, particularly in subcortical regions where clearance is less efficient (Voumvourakis et al., 2023; Wang & Holtzman, 2020). These pathological changes can damage the white matter tracts within the subcortical regions, thereby disrupting SFC. Notably, glymphatic dysfunction has been linked to tau accumulation predominantly in subcortical regions, rather than cortical areas, with this subcortical tau burden mediating the relationship between clearance impairment and clinical severity in neurodegenerative conditions (Hsu et al., 2024). Similarly, acute sleep deprivation has been shown to increase amyloid-β accumulation in subcortical regions such as the hippocampus and thalamus, negatively correlating with total sleep time (Shokri-Kojori et al., 2018).

These findings suggest a plausible mechanism through which impaired glymphatic clearance, as a consequence of poor sleep, may contribute to WM deficits by affecting subcortical white matter pathways and disrupting the SFCs they support. In this way, our results highlight a potential sleep–glymphatic–SFC pathway that may underlie cognitive impairments in ID patients. Nevertheless, interactive or bidirectional relationships between glymphatic function and SFC remain biologically plausible. One possibility is that glymphatic clearance and SFC form a mutually constraining system: impaired clearance may increase metabolic burden and compromise white-matter–dependent communication, whereas altered coupling may also relate to structural vulnerability in regions where clearance is less efficient. This perspective implies that glymphatic function could *moderate* the cognitive consequences of network coupling (or vice versa), rather than acting solely as an upstream mediator. However, because the data are cross-sectional, this pathway should be interpreted as an associational pattern rather than a causal sequence, and the temporal directionality among sleep quality, DTI-ALPS, SFC, and cognition cannot be definitively established. Future studies employing longitudinal designs, experimental manipulations, or both can directly test potential interaction effects and clarify the temporal ordering of these processes.

### Limitations

This study has several limitations that should be noted. First, the cross-sectional design limits the ability to draw causal inferences. Although mediation models can characterize statistical associations, they cannot establish temporal precedence or determine whether alterations in DTI-ALPS or SFC directly contribute to memory deficits. Future longitudinal or sleep-manipulation studies will be necessary to clarify the causal directionality among sleep quality, glymphatic dynamics, SFC, and cognitive performance. Second, the study lacked a healthy control group. Without a comparison sample, it remains unclear whether the observed levels of glymphatic function, SFC, or WM performance in ID patients are significantly altered or pathological. This absence limits the interpretability of group-level abnormalities and prevents inferences regarding the relative impairment of glymphatic dynamics in insomnia disorder. Moreover, because the glymphatic system remains insufficiently characterized in human neuroscience (Voumvourakis et al., 2023), and DTI-ALPS is an indirect proxy rather than a direct measure of glymphatic dynamics (Tzschätzsch et al., 2018), the biological mechanisms underlying the observed associations cannot be specified with certainty. In particular, DTI-ALPS does not allow us to determine whether the links to cognition reflect direct effects or indirect pathways related to homeostatic regulation and neuroinflammation. Accordingly, mechanistic interpretations should be made with caution, and future work combining complementary glymphatic-sensitive measures with longitudinal or experimental designs will be important for clarifying these pathways. Third, WM was assessed using the digit span backward task, which may primarily reflect short-term memory capacity rather than dynamic WM processes (St Clair-Thompson, 2010). Future studies should consider established paradigms like the N-back task for validation. Fourth, sleep quality was measured solely through self-report, which may be affected by individual biases and emotional states (Pierson-Bartel & Ujma, 2024) and, therefore, may attenuate the strength of brain–behavior associations observed in this study. The absence of objective sleep measures such as actigraphy or polysomnography also limits our ability to determine how specific physiological aspects of sleep, such as total sleep time, sleep efficiency, slow-wave activity, or arousal burden, relate to glymphatic function and SFC. Future studies incorporating both subjective questionnaires and objective sleep assessments would provide a more comprehensive characterization of sleep disturbances and strengthen the interpretability and generalizability of sleep–brain relationships in ID. Finally, glymphatic function was estimated using DTI-ALPS indices from the lateral ventricles. Although previous work supports this approach (Zhang et al., 2021), regional heterogeneity in glymphatic pathways (McKnight et al., 2021) means that lateral ventricular measures may not fully capture whole-brain glymphatic dynamics.

## Conclusion

This study provides novel evidence linking reduced DTI-ALPS and disrupted SFC to WM deficits in patients with insomnia disorder. Using multimodal neuroimaging, we demonstrate that poorer sleep quality is associated with reduced DTI-ALPS, which in turn correlates with lower SFC within the subcortical network and diminished WM performance. Importantly, these two neurophysiological mechanisms jointly mediate the effect of sleep disturbance on WM. Our findings highlight a network-specific, biologically plausible pathway through which disrupted sleep physiology contributes to domain-specific cognitive impairments. These results underscore the clinical value of DTI-ALPS and SFC as potential neuroimaging biomarkers for identifying insomnia-related cognitive vulnerability.

## Supporting information

10.1017/S0033291726104188.sm001 Lv et al. supplementary material Lv et al. supplementary material

## Supplementary material

The supplementary material for this article can be found at http://doi.org/10.1017/S0033291726104188.

## Long descriptions

### Figure 1. Long description

The flowchart is divided into five main sections.

Panel A, Data collection process, shows a linear sequence of icons from left to right. It begins with semi-structured interviews, followed by a group representing insomnia disorder patients, a P S Q I questionnaire, a cognitive test, and ends with M R I scanning.

Panel B, Multimodal neuroimaging Calculation, is split into two sub-sections. On the left, r s f M R I data undergoes Pearson correlation to create functional connectivity maps, while d M R I data uses deterministic tracking and a Brainnetome atlas to create structural connectivity maps. On the right, Structural-functional coupling is shown as a scatter plot of functional versus structural connectivity, linked to Yeo’s R S N and subcortical regions. Below this, Glymphatic function D T I A L P S is calculated from a brain cross-section at z equals 32, showing regions of interest. A diagram illustrates the perivascular space with blue projection fibers and green association fibers.

Panel C, Neural correlates of D T I A L P S, shows a comparison between D T I A L P S and brain networks leading to a circular diagram with three overlapping segments.

Panel D, Neural correlates of cognition function, shows arrows from D T I A L P S and S F C brain maps pointing toward a central icon of a person performing a cognitive test.

Panel E, Neural mechanism of working memory, is a triangular path diagram. P S Q I points to A L P S forward slash S F C and W M. A L P S forward slash S F C also points down to W M, representing the integrated neurocognitive mechanism.

Navigate back to Figure 1..

### Table 1. Long description

The table is organized into five columns: Characteristic, Number, M plus or minus S D or n percentage, Minimum, and Maximum.

Demographic information:

* Age in years: Number 391, Mean 43.15 plus or minus 15.39, Minimum 18.42, Maximum 75.33.

* Gender, women: Number 391, 224 (57.29 percent).

* Handedness, left-handed: Number 391, 8 (2.04 percent).

Pittsburgh Sleep Quality Index (P S Q I):

* Total scores: Number 265, Mean 11.63 plus or minus 3.47, Minimum 5, Maximum 21.

* Sleep quality: Number 265, Mean 2.48 plus or minus 0.64, Minimum 1, Maximum 3.

* Sleep latency: Number 265, Mean 2.11 plus or minus 0.86, Minimum 0, Maximum 3.

* Sleep duration: Number 265, Mean 1.21 plus or minus 1.18, Minimum 0, Maximum 3.

* Sleep efficiency: Number 265, Mean 1.1 plus or minus 1.12, Minimum 0, Maximum 3.

* Sleep disturbances: Number 265, Mean 1.49 plus or minus 0.6, Minimum 0, Maximum 3.

* Sleep medication: Number 265, Mean 0.82 plus or minus 1.22, Minimum 0, Maximum 3.

* Daytime dysfunction: Number 265, Mean 2.42 plus or minus 0.81, Minimum 0, Maximum 3.

Subjective sleep indicators:

* Total sleep time in hours: Number 332, Mean 7.11 plus or minus 1.72, Minimum 1.11, Maximum 16.85.

* Sleep onset latency in hours: Number 332, Mean 0.40 plus or minus 0.49, Minimum 0.01, Maximum 4.2.

* Sleep efficiency percentage: Number 332, Mean 92.57 plus or minus 7.86, Minimum 31.9, Maximum 99.94.

Navigate back to Table 1..

### Figure 2. Long description

The graphic is divided into four panels labeled A through D.

Panel A, titled Region of interest, shows an axial brain slice at Z equals 32. Two regions are highlighted bilaterally. The superior longitudinal fasciculus is colored green and the superior or posterior corona radiata is colored blue.

Panel B, titled Perivascular space, is a schematic diagram. It shows two horizontal gray cylinders representing the perivascular space. Three vertical blue arrows labeled Projection fiber intersect the cylinders. Three diagonal green cylinders labeled Association fiber pass through the perivascular space at an angle. A three-dimensional coordinate system at the bottom right shows the x, y, and z axes.

Panel C, titled Correlation between bilateral A L P S, is a scatter plot with marginal density plots. The x-axis is Right D T I A L P S and the y-axis is Left D T I A L P S. The data points show a strong linear increase with a correlation coefficient r equals 0.89 and a p-value less than 0.001.

Panel D, titled Correlation between A L P S and P S Q I, is a scatter plot. The x-axis is P S Q I ranging from 5 to 20 and the y-axis is D T I A L P S ranging from 1.4 to 1.8. The data points show a slight linear decrease with a correlation coefficient r equals negative 0.17 and a p-value of 0.006.

Navigate back to Figure 2..

### Figure 3. Long description

A grid of nine scatter plots arranged in three rows and three columns. Each plot includes a regression line with a shaded confidence interval, a horizontal dashed red line at zero, and marginal density plots in green on the top and orange on the right.

* Panel A: Global S F C versus D T I A L P S shows a positive correlation with r equals 0.32 and p F D R less than 0.001.

* Panel B: V N S F C versus D T I A L P S shows a positive correlation with r equals 0.20 and p F D R less than 0.001.

* Panel C: L N S F C versus D T I A L P S shows a negative correlation with r equals negative 0.12 and p F D R equals 0.014.

* Panel D: Global S F C versus T E M L shows a flat trend with r equals 0.01 and p F D R equals 0.84.

* Panel E: V N S F C versus T E M L shows a flat trend with r equals negative 0.02 and p F D R equals 0.77.

* Panel F: L N S F C versus T E M L shows a flat trend with r equals 0.02 and p F D R equals 0.80.

* Panel G: Sub S F C versus D T I A L P S shows a positive correlation with r equals 0.29 and p F D R less than 0.001.

* Panel H: Sub S F C versus T E M L shows a positive correlation with r equals 0.28 and p F D R less than 0.001.

* Panel I: D T I A L P S versus T E M L shows a positive correlation with r equals 0.30 and p F D R less than 0.001.

The x-axes for the first and third columns are D T I A L P S and T E M L respectively. The y-axes vary by row, representing global, network-specific, or subcortical structural-functional coupling.

Navigate back to Figure 3..

### Figure 4. Long description

The image contains three panels labeled A, B, and C.

Panel A, Mediation model of glymphatic function. A triangular path diagram shows P S Q I on the bottom left and T E M L on the bottom right. A direct arrow from P S Q I to T E M L has coefficients c equals negative 0.203 triple asterisk and c prime equals negative 0.155 double asterisk. Above them is A L P S. An arrow from P S Q I to A L P S is labeled a equals negative 0.244 double asterisk. An arrow from A L P S to T E M L is labeled b equals 0.198 triple asterisk. Below the diagram, Indirect 95 percent C I equals negative 0.086 to negative 0.017.

Panel B, Mediation model of subcortical network. The structure is identical to Panel A but the mediator is Sub S F C. The path from P S Q I to Sub S F C is a equals negative 0.240 triple asterisk. The path from Sub S F C to T E M L is b equals 0.191 triple asterisk. The direct path remains c equals negative 0.203 triple asterisk and c prime equals negative 0.157 double asterisk. Below the diagram, Indirect 95 percent C I equals negative 0.090 to negative 0.012.

Panel C, Serial mediation model of working memory. This model combines the variables. P S Q I is on the left and T E M L is on the right. Above them are two mediators, A L P S followed by Sub S F C.

- Direct path from P S Q I to T E M L: c equals negative 0.203 triple asterisk, c prime equals negative 0.129 asterisk.

- Path from P S Q I to A L P S: a sub 1 equals negative 0.244 double asterisk.

- Path from P S Q I to Sub S F C: a sub 2 equals negative 0.182 double asterisk.

- Path from A L P S to Sub S F C: d equals 0.237 triple asterisk.

- Path from A L P S to T E M L: b sub 1 equals 0.164 triple asterisk.

- Path from Sub S F C to T E M L: b sub 2 equals 0.143 double asterisk.

Below the diagram, Indirect 95 percent C I equals negative 0.127 to negative 0.030.

Navigate back to Figure 4..
