# Supplementary material for: DTI-ALPS and subcortical structural-functional coupling mediate the impact of sleep quality on working memory in insomnia disorder
Source: Psychol Med. 2026 Jun 19;56:e201. doi: 10.1017/S0033291726104188 (PMC13280695; doi:10.1017/S0033291726104188)
Supplement: Lv et al. supplementary material [file S0033291726104188sup001.docx]

DTI-ALPS and Subcortical Structural-Functional Coupling Mediate the Impact of Sleep Quality on Working Memory in Insomnia Disorder

Zhangwei Lv^1, 2^, Haobo Zhang^1, 2^, Yinian Yang^1, 2^, Yuxian Wei^1, 2^, Xu Lei^1,2, *^

^1^Sleep and NeuroImaging Center, Faculty of Psychology, Southwest University, Chongqing, 400715, China

*^2^Key Laboratory of Cognition and Personality (Southwest University), Ministry of Education, Chongqing, 400715, China*

***Corresponding author**, E-mail: xlei@swu.edu.cn. Telephone number: +86 023-68252983.

Address: Sleep and NeuroImaging Center, Faculty of Psychology, Southwest University, Chongqing 400715, China

***Corresponding author**: Zhangwei Lv, PhD, E-mail: lvzhangwei@aliyun.com. Telephone number: +86 15518177783.

Address: Sleep and NeuroImaging Center, Faculty of Psychology, Southwest University, Chongqing 400715, China

Table S1. Samples of the study, consortium sites, sample size data acquisition parameters.

| Dataset | Sites | Scanner | Numbers | T1w Parameters | dMRI Parameters | rs-fMRI Parameters |
| --- | --- | --- | --- | --- | --- | --- |
| SWU dataset | Southwest University | 3.0 Tesla Siemens Prisma MRI Scanner | 112 | 64 channel, TR/TE = 2530/2.98 ms, Flip Angle = 7^o^, Slice number = 192, Voxel size = 0.5 × 0.5 × 1 mm^3^, FOV read = 256 mm, phase enc.dir = A >> P, acquisition matrix = 512 × 512 | TR/TE = 2400ms/71 ms, Slices number = 76, Voxel size = 2 × 2 × 2 mm^3^, FoV read = 224 mm, Phase enc.dir = P >> A, b value = 0/1000/2000 s/mm^2^, Base resolution = 112, Diff direction = 64 | TR/TE = 2000/30 ms, Flip Angle = 90°, Slice number = 62, voxel size = 3 × 3 × 3 mm^3^, FOV = 224mm, phase enc.dir = P >> A, acquisition matrix = 112×112, Time points = 240 |
| UMI dataset | Chongqing United Medical Imaging | 3.0T GE SIGNA Pioneer MRI Scanner | 319 | 64 channel, TR/TE = 2530/2.98 ms, Flip Angle = 7^o^, Slice number = 192, Voxel size = 0.5 × 0.5 × 1 mm^3^, FOV read = 256 mm, phase enc.dir = A >> P, acquisition matrix = 512 × 512 | TR/TE = 2400ms/71 ms, Slices number = 76, Voxel size = 2 × 2 × 2 mm^3^, FoV read = 224 mm, Phase enc.dir = P >> A, b value = 0/1000 s/mm^2^, Base resolution = 112, Diff direction = 64 | TR/TE = 2000/30 ms, Flip Angle = 90°, Slice number***** = [33~36], voxel size = 2 × 2 × 2 mm^3^, FOV = 224 mm, phase enc.dir = P >> A, Time points = 240 |

- **Sites:** Brain Imaging Data Scanning Locations; * Slice number was adjusted (33–36 slices) to accommodate individual head sizes.

MRI preprocessing

The rs-fMRI preprocessing process included the initial 10 volumes were eliminated, slice timing, realignment, head motion correction, normalization to the Montreal Neurological Institute (MNI) coordinate space with 2 × 2 × 2 mm^3^, and band-pass filtering (0.01-0.1 Hz). Additionally, the process involved regressing out interfering signals, including the Friston 24 model of head motion parameters (Friston, Williams, Howard, Frackowiak, & Turner, 1996), as well as signals from global brain activity, white matter, and cerebrospinal fluid.

The dMRI preprocessing was conducted using the Pipeline for Analyzing braiN Diffusion images (PANDA) toolbox (Cui, Zhong, Xu, He, & Gong, 2013). Specifically, the original dMRI data were first resampled to 2 × 2 × 2 mm³ to reduce computational burden and improve processing efficiency. The dMRI data format was then converted from DICOM to NIfTI for further analysis. During preprocessing, we extracted one b = 0 image and 64 b = 1000 images from the dataset (the SWU dataset was collected with b = 0, 1000, and 2000 s/mm², whereas the UMI dataset included b = 0 and 1000 s/mm².). Skull stripping was then performed on the b = 0 image, generating a brain mask with the skull removed. To reduce storage costs and enhance processing speed, we applied cropping gap, replacing the original image with a smaller image that retained only brain tissue. The cropping gap was defined as the distance (3 mm) between the selected image boundary and the brain boundary.

Next, eddy current correction and motion correction were performed to minimize artifacts. Finally, we computed DTI metrics, including fractional anisotropy (FA), mean diffusivity (MD), radial diffusivity (RD/L23m), and diffusivity along the x, y, and z directions, and registered them to the T1w images for spatial alignment.

Table S2. Node Information and Network Affiliation in the Brainnetome Atlas (Fan et al., 2016)

| **Lobe** | **Gyrus** | **Left and Right Hemisphere** | **Label ID.L** | **Label ID.R** | **lh network*** | **rh network** |
| --- | --- | --- | --- | --- | --- | --- |
| **Frontal Lobe** | SFG, Superior Frontal Gyrus | SFG_L(R)_7_1 | 1 | 2 | 6 | 4 |
|  |  | SFG_L(R)_7_2 | 3 | 4 | 7 | 6 |
|  |  | SFG_L(R)_7_3 | 5 | 6 | 7 | 7 |
|  |  | SFG_L(R)_7_4 | 7 | 8 | 3 | 3 |
|  |  | SFG_L(R)_7_5 | 9 | 10 | 2 | 2 |
|  |  | SFG_L(R)_7_6 | 11 | 12 | 7 | 6 |
|  |  | SFG_L(R)_7_7 | 13 | 14 | 7 | 7 |
|  | MFG, Middle Frontal Gyrus | MFG_L(R)_7_1 | 15 | 16 | 4 | 6 |
|  |  | MFG_L(R)_7_2 | 17 | 18 | 6 | 6 |
|  |  | MFG_L(R)_7_3 | 19 | 20 | 6 | 6 |
|  |  | MFG_L(R)_7_4 | 21 | 22 | 6 | 6 |
|  |  | MFG_L(R)_7_5 | 23 | 24 | 7 | 6 |
|  |  | MFG_L(R)_7_6 | 25 | 26 | 3 | 3 |
|  |  | MFG_L(R)_7_7 | 27 | 28 | 5 | 6 |
|  | IFG, Inferior Frontal Gyrus | IFG_L(R)_6_1 | 29 | 30 | 6 | 3 |
|  |  | IFG_L(R)_6_2 | 31 | 32 | 6 | 6 |
|  |  | IFG_L(R)_6_3 | 33 | 34 | 7 | 7 |
|  |  | IFG_L(R)_6_4 | 35 | 36 | 7 | 6 |
|  |  | IFG_L(R)_6_5 | 37 | 38 | 4 | 4 |
|  |  | IFG_L(R)_6_6 | 39 | 40 | 4 | 4 |
|  | OrG, Orbital Gyrus | OrG_L(R)_6_1 | 41 | 42 | 7 | 7 |
|  |  | OrG_L(R)_6_2 | 43 | 44 | 7 | 7 |
|  |  | OrG_L(R)_6_3 | 45 | 46 | 5 | 6 |
|  |  | OrG_L(R)_6_4 | 47 | 48 | 5 | 5 |
|  |  | OrG_L(R)_6_5 | 49 | 50 | 5 | 5 |
|  |  | OrG_L(R)_6_6 | 51 | 52 | 7 | 7 |
|  | PrG, Precentral Gyrus | PrG_L(R)_6_1 | 53 | 54 | 2 | 2 |
|  |  | PrG_L(R)_6_2 | 55 | 56 | 3 | 3 |
|  |  | PrG_L(R)_6_3 | 57 | 58 | 2 | 2 |
|  |  | PrG_L(R)_6_4 | 59 | 60 | 2 | 2 |
|  |  | PrG_L(R)_6_5 | 61 | 62 | 4 | 4 |
|  |  | PrG_L(R)_6_6 | 63 | 64 | 3 | 3 |
|  | PCL, Paracentral Lobule | PCL_L(R)_2_1 | 65 | 66 | 4 | 2 |
|  |  | PCL_L(R)_2_2 | 67 | 68 | 2 | 2 |
| **Temporal Lobe** | STG, Superior Temporal Gyrus | STG_L(R)_6_1 | 69 | 70 | 5 | 5 |
|  |  | STG_L(R)_6_2 | 71 | 72 | 2 | 2 |
|  |  | STG_L(R)_6_3 | 73 | 74 | 2 | 2 |
|  |  | STG_L(R)_6_4 | 75 | 76 | 2 | 2 |
|  |  | STG_L(R)_6_5 | 77 | 78 | 5 | 5 |
|  |  | STG_L(R)_6_6 | 79 | 80 | 7 | 7 |
|  | MTG, Middle Temporal Gyrus | MTG_L(R)_4_1 | 81 | 82 | 7 | 6 |
|  |  | MTG_L(R)_4_2 | 83 | 84 | 7 | 7 |
|  |  | MTG_L(R)_4_3 | 85 | 86 | 3 | 3 |
|  |  | MTG_L(R)_4_4 | 87 | 88 | 7 | 7 |
|  | ITG, Inferior Temporal Gyrus | ITG_L(R)_7_1 | 89 | 90 | 5 | 5 |
|  |  | ITG_L(R)_7_2 | 91 | 92 | 3 | 3 |
|  |  | ITG_L(R)_7_3 | 93 | 94 | 5 | 5 |
|  |  | ITG_L(R)_7_4 | 95 | 96 | 7 | 5 |
|  |  | ITG_L(R)_7_5 | 97 | 98 | 3 | 3 |
|  |  | ITG_L(R)_7_6 | 99 | 100 | 6 | 6 |
|  |  | ITG_L(R)_7_7 | 101 | 102 | 5 | 5 |
|  | FuG, Fusiform Gyrus | FuG_L(R)_3_1 | 103 | 104 | 5 | 5 |
|  |  | FuG_L(R)_3_2 | 105 | 106 | 1 | 1 |
|  |  | FuG_L(R)_3_3 | 107 | 108 | 3 | 1 |
|  | PhG, Parahippocampal Gyrus | PhG_L(R)_6_1 | 109 | 110 | 5 | 5 |
|  |  | PhG_L(R)_6_2 | 111 | 112 | 5 | 1 |
|  |  | PhG_L(R)_6_3 | 113 | 114 | 1 | 1 |
|  |  | PhG_L(R)_6_4 | 115 | 116 | 5 | 5 |
|  |  | PhG_L(R)_6_5 | 117 | 118 | 5 | 5 |
|  |  | PhG_L(R)_6_6 | 119 | 120 | 1 | 1 |
|  | pSTS, posterior Superior Temporal Sulcus | pSTS_L(R)_2_1 | 121 | 122 | 7 | 7 |
|  |  | pSTS_L(R)_2_2 | 123 | 124 | 4 | 4 |
| **Parietal Lobe** | SPL, Superior Parietal Lobule | SPL_L(R)_5_1 | 125 | 126 | 3 | 3 |
|  |  | SPL_L(R)_5_2 | 127 | 128 | 3 | 3 |
|  |  | SPL_L(R)_5_3 | 129 | 130 | 3 | 3 |
|  |  | SPL_L(R)_5_4 | 131 | 132 | 2 | 2 |
|  |  | SPL_L(R)_5_5 | 133 | 134 | 3 | 3 |
|  | IPL, Inferior Parietal Lobule | IPL_L(R)_6_1 | 135 | 136 | 1 | 1 |
|  |  | IPL_L(R)_6_2 | 137 | 138 | 6 | 6 |
|  |  | IPL_L(R)_6_3 | 139 | 140 | 3 | 3 |
|  |  | IPL_L(R)_6_4 | 141 | 142 | 7 | 6 |
|  |  | IPL_L(R)_6_5 | 143 | 144 | 3 | 7 |
|  |  | IPL_L(R)_6_6 | 145 | 146 | 2 | 2 |
|  | Pcun, Precuneus | PCun_L(R)_4_1 | 147 | 148 | 6 | 6 |
|  |  | PCun_L(R)_4_2 | 149 | 150 | 2 | 3 |
|  |  | PCun_L(R)_4_3 | 151 | 152 | 1 | 1 |
|  |  | PCun_L(R)_4_4 | 153 | 154 | 7 | 7 |
|  | PoG, Postcentral Gyrus | PoG_L(R)_4_1 | 155 | 156 | 2 | 2 |
|  |  | PoG_L(R)_4_2 | 157 | 158 | 2 | 2 |
|  |  | PoG_L(R)_4_3 | 159 | 160 | 3 | 2 |
|  |  | PoG_L(R)_4_4 | 161 | 162 | 2 | 2 |
| **Insular Lobe** | INS, Insular Gyrus | INS_L(R)_6_1 | 163 | 164 | 2 | 2 |
|  |  | INS_L(R)_6_2 | 165 | 166 | 8 | 6 |
|  |  | INS_L(R)_6_3 | 167 | 168 | 4 | 4 |
|  |  | INS_L(R)_6_4 | 169 | 170 | 4 | 4 |
|  |  | INS_L(R)_6_5 | 171 | 172 | 2 | 2 |
|  |  | INS_L(R)_6_6 | 173 | 174 | 4 | 4 |
| **Limbic Lobe** | CG, Cingulate Gyrus | CG_L(R)_7_1 | 175 | 176 | 7 | 7 |
|  |  | CG_L(R)_7_2 | 177 | 178 | 8 | 8 |
|  |  | CG_L(R)_7_3 | 179 | 180 | 7 | 4 |
|  |  | CG_L(R)_7_4 | 181 | 182 | 7 | 1 |
|  |  | CG_L(R)_7_5 | 183 | 184 | 4 | 4 |
|  |  | CG_L(R)_7_6 | 185 | 186 | 4 | 4 |
|  |  | CG_L(R)_7_7 | 187 | 188 | 7 | 7 |
| **Occipital Lobe** | MVOcC*,* MedioVentral Occipital Cortex | MVOcC _L(R)_5_1 | 189 | 190 | 1 | 1 |
|  |  | MVOcC _L(R)_5_2 | 191 | 192 | 1 | 1 |
|  |  | MVOcC _L(R)_5_3 | 193 | 194 | 1 | 1 |
|  |  | MVOcC _L(R)_5_4 | 195 | 196 | 1 | 1 |
|  |  | MVOcC _L(R)_5_5 | 197 | 198 | 1 | 1 |
|  | LOcC, lateral Occipital Cortex | LOcC_L(R)_4_1 | 199 | 200 | 1 | 1 |
|  |  | LOcC _L(R)_4_2 | 201 | 202 | 3 | 1 |
|  |  | LOcC _L(R)_4_3 | 203 | 204 | 1 | 1 |
|  |  | LOcC_L(R)_4_4 | 205 | 206 | 1 | 1 |
|  |  | LOcC _L(R)_2_1 | 207 | 208 | 1 | 1 |
|  |  | LOcC _L(R)_2_2 | 209 | 210 | 1 | 1 |
| **Subcortical Nuclei** | Amyg, Amygdala | Amyg_L(R)_2_1 | 211 | 212 | 8 | 8 |
|  |  | Amyg_L(R)_2_2 | 213 | 214 | 8 | 8 |
|  | Hipp, Hippocampus | Hipp_L(R)_2_1 | 215 | 216 | 8 | 8 |
|  |  | Hipp_L(R)_2_2 | 217 | 218 | 8 | 8 |
|  | BG, Basal Ganglia | BG_L(R)_6_1 | 219 | 220 | 8 | 8 |
|  |  | BG_L(R)_6_2 | 221 | 222 | 8 | 8 |
|  |  | BG_L(R)_6_3 | 223 | 224 | 8 | 8 |
|  |  | BG_L(R)_6_4 | 225 | 226 | 8 | 8 |
|  |  | BG_L(R)_6_5 | 227 | 228 | 8 | 8 |
|  |  | BG_L(R)_6_6 | 229 | 230 | 8 | 8 |
|  | Tha, Thalamus | Tha_L(R)_8_1 | 231 | 232 | 8 | 8 |
|  |  | Tha_L(R)_8_2 | 233 | 234 | 8 | 8 |
|  |  | Tha_L(R)_8_3 | 235 | 236 | 8 | 8 |
|  |  | Tha_L(R)_8_4 | 237 | 238 | 8 | 8 |
|  |  | Tha_L(R)_8_5 | 239 | 240 | 8 | 8 |
|  |  | Tha_L(R)_8_6 | 241 | 242 | 8 | 8 |
|  |  | Tha_L(R)_8_7 | 243 | 244 | 8 | 8 |
|  |  | Tha_L(R)_8_8 | 245 | 246 | 8 | 8 |

* The network classification in **Table S2** is based on the **Yeo 7-network parcellation**, which includes the **Visual (1), Somatomotor (2), Dorsal Attention (3), Ventral Attention (4), Limbic (5), Frontoparietal (6), and Default Mode (7) networks (Yeo et al., 2011).** Additionally, **a subcortical network (8)** is included to account for subcortical structures.

Table S3. Within-site variance before vs. after ComBat harmonization

| **Metric** | **Mean Variance (Pre)** | **Mean Variance (Post)** | **Δ (Post – Pre)** | ***t*** | ***p*** |
| --- | --- | --- | --- | --- | --- |
| **SFC** | 0.162 | 0.162 | −0.000181 | 0.61 | 0.539 |
| **FC** | 0.064 | 0.061 | −0.002583 | 114.90 | < 0.001 |
| **SC** | 7.187 | 7.110 | −0.077227 | 1.41 | 0.158 |

Table S4. Model comparison statistics for the three mediation models

| **Model** | **Path Structure** | **AIC** | **BIC** | **R²**  **(Mediator 1)** | **R²**  **(Mediator 2)** | **R²**  **(TEML)** |
| --- | --- | --- | --- | --- | --- | --- |
| Model 1 | PSQI → DTI-ALPS → TEML | 1032.751 | 1055.84 | DTI-ALPS = 0.048 | — | 0.144 |
| Model 2 | PSQI → Sub-SFC → TEML | 994.41 | 1017.498 | Sub-SFC = 0.057 | — | 0.125 |
| Model 3 | PSQI → DTI-ALPS → Sub-SFC → TEML (serial mediation) | 1579.52 | 1615.802 | DTI-ALPS = 0.048 | Sub-SFC = 0.123 | 0.125 |


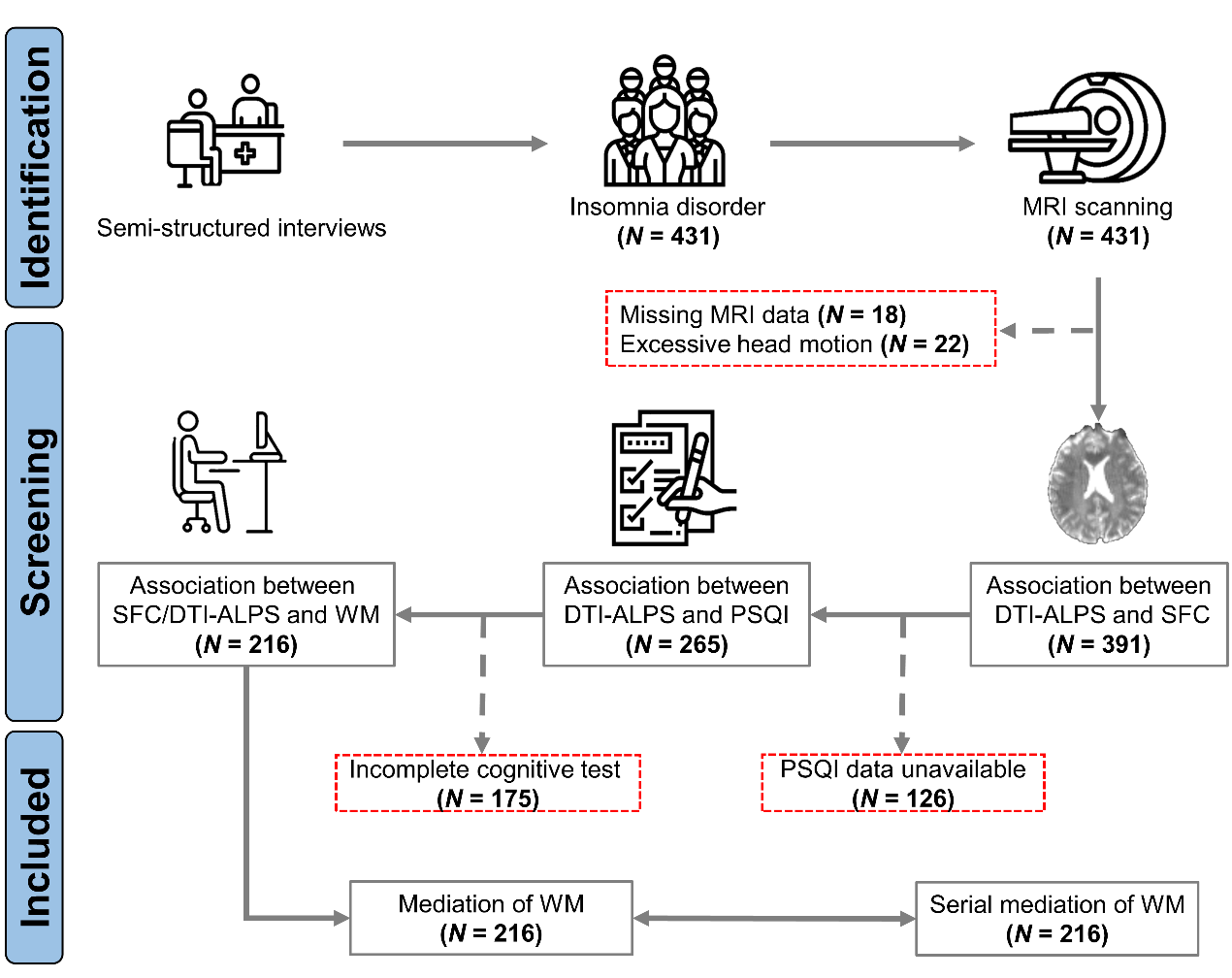


**Figure S1. Participant enrollment and exclusion flow.** Non-responders: did not complete the PSQI.


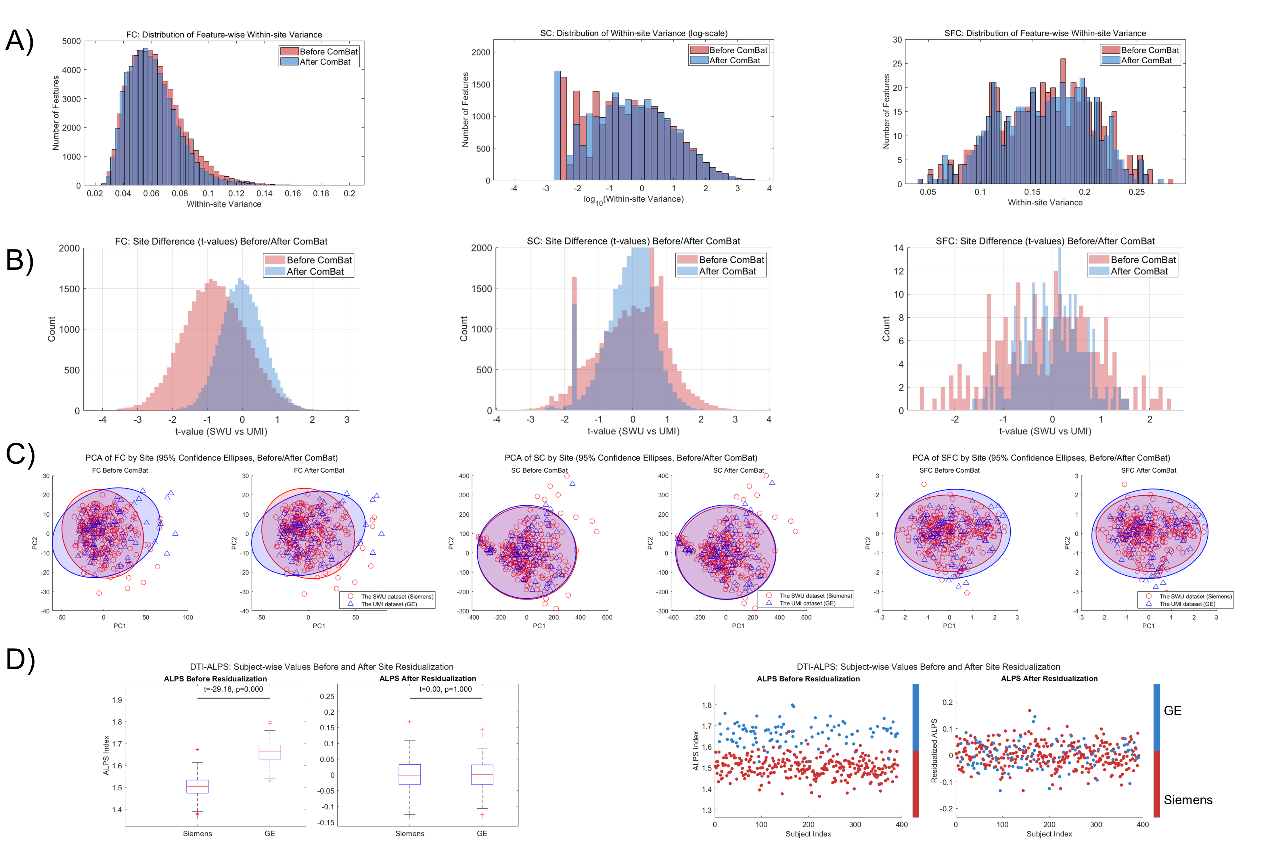


**Figure S2. Evaluation of site effects and ComBat harmonization across SC, FC, SFC, and DTI-ALPS. (A)** Within-site variance distributions before and after ComBat harmonization. Histograms show the feature-wise within-site variance for SC, FC, and SFC matrices across the two scanning sites (Siemens vs. GE). FC exhibited a marked reduction in site-related variance after harmonization, whereas SC and SFC showed smaller reductions. **(B)** Distribution of site differences before and after harmonization. Overlaid histograms display the t-value distributions for SC, FC, and SFC when comparing the two sites (SWU vs. UMI). After ComBat, the distributions shift substantially toward zero—most prominently for FC—indicating reduced scanner-related bias. **(C)** PCA visualization of multivariate site effects. Principal component analysis (PCA) was applied to SFC matrices before and after harmonization. Scatter plots show participants projected onto the first two principal components, with points colored by site. Ellipses represent 95% confidence intervals. Before ComBat, partial site separation is observed, whereas after harmonization, the two sites exhibit substantial overlap, indicating effective reduction of multivariate site structure. **(D)** DTI-ALPS before and after residualization for site. Boxplots compare the DTI-ALPS index between sites before and after regressing out site, age, and sex. A significant site difference was present prior to adjustment but disappeared after residualization. The accompanying scatter plots show subject-wise DTI-ALPS values colored by site, further illustrating the removal of site effects.


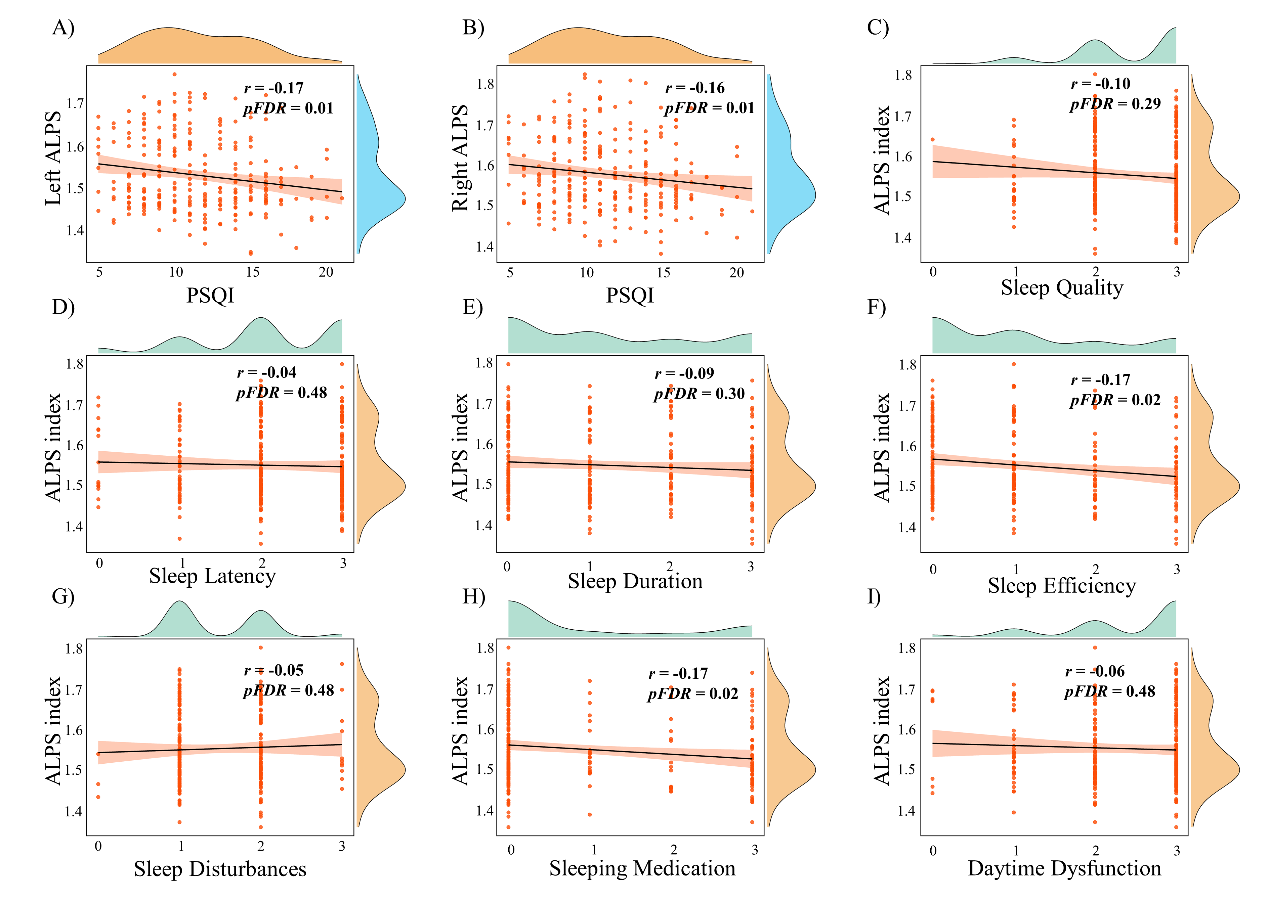


**Figure S3.** **Correlations between DTI-ALPS index and PSQI measures.** **A–B)** Scatter plots showing associations between **(A)** left DTI-ALPS index and PSQI total score (r = –0.18, pFDR = 0.01) and **(B)** right DTI-ALPS index and PSQI total score (r = –0.16, pFDR = 0.01). **C–I)** Partial correlation analyses (FDR-corrected) between DTI-ALPS index and seven PSQI subcomponents. Significant negative correlations were observed for **(C)** sleep efficiency (r = –0.17, pFDR = 0.02) and **(D)** sleep medication use (r = –0.17, pFDR = 0.02). No other PSQI subcomponents showed significant FDR-corrected associations with DTI-ALPS index.


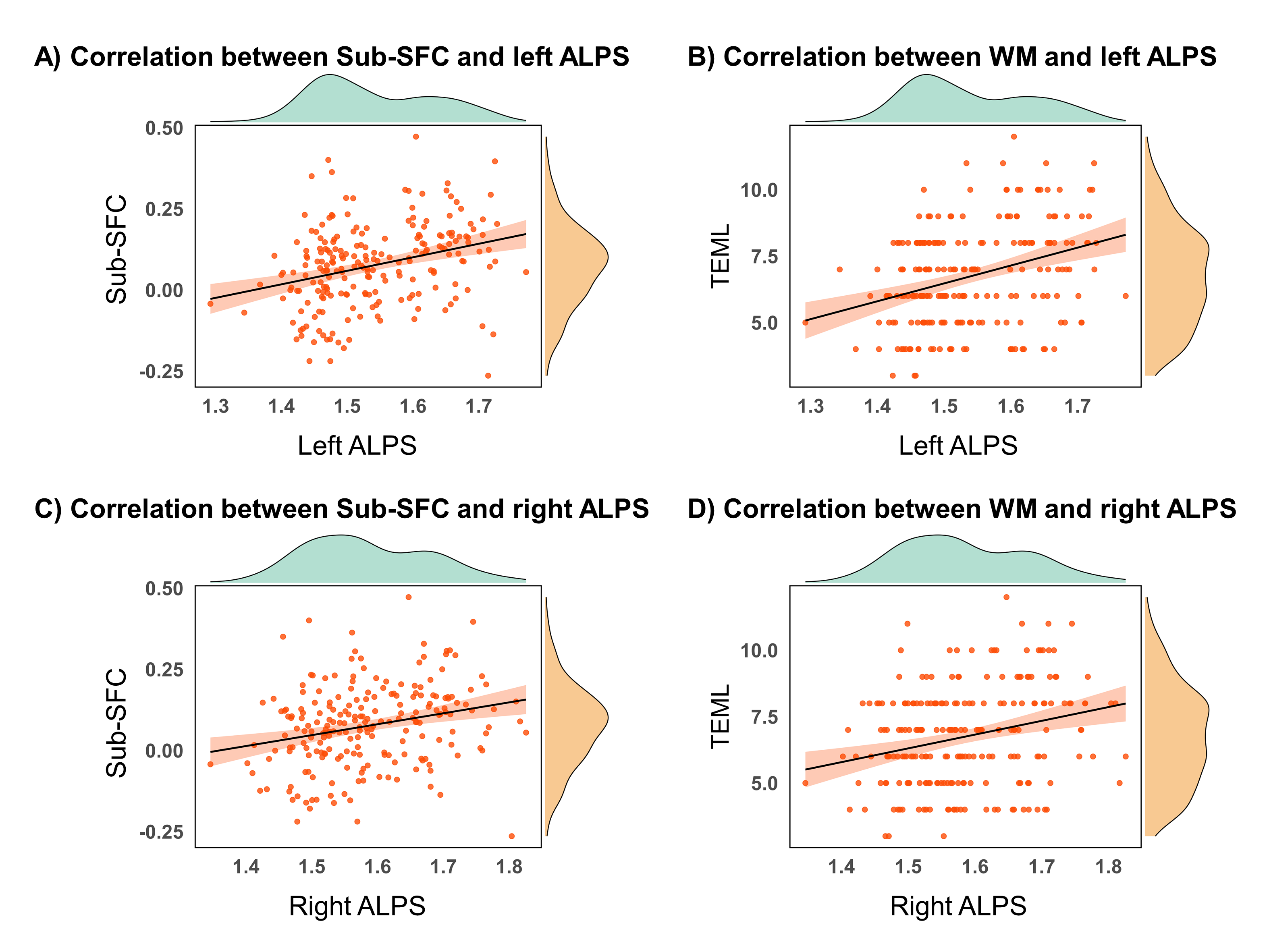


**Figure S4.** **This figure illustrates the correlations between left and right DTI-ALPS indices and two major outcome measures: Sub-SFC and WM performance (TEML).** Both left and right DTI-ALPS showed significant positive correlations with working memory (left: r = 0.33, p*FDR* < 0.001; right: r = 0.26, p*FDR* < 0.001) as well as significant positive associations with Sub-SFC (left: r = 0.31, p*FDR* < 0.001; right: r = 0.28, p*FDR* < 0.001).


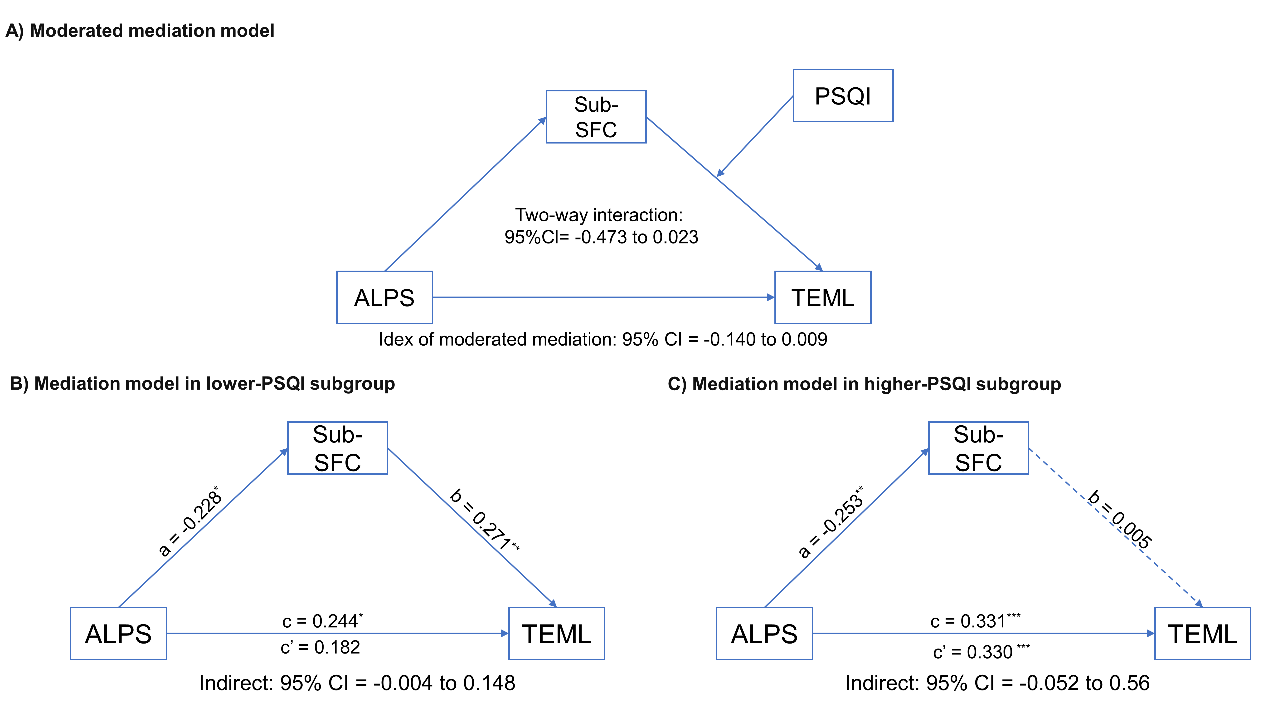


**Figure S5. Moderated mediation analysis examining whether PSQI-defined sleep-disturbance severity alters the association between Sub-SFC and WM.** **A) Moderated mediation model.** This model tested whether the association between Sub-SFC and TEML differs across levels of sleep disturbance, indexed by PSQI-based subgroups. The interaction between Sub-SFC and the PSQI subgroup on TEML showed a trend in the expected direction but did not reach conventional significance (*β* = –0.225, *p* = 0.076; interaction 95% CI = –0.473 to 0.023). The index of moderated mediation was similarly trend-level (index = –0.058, bootstrapped 95% CI [–0.140, 0.009]), suggesting that the relationship between Sub-SFC and WM may vary with the severity of sleep disturbance. **B) Mediation model in the lower-PSQI subgroup.** Among participants with relatively lower PSQI scores, Sub-SFC significantly mediated the association between DTI-ALPS and WM. The indirect effect via Sub-SFC was significant (*β* = 0.062, SE = 0.038, bootstrapped 95% CI [0.004, 0.148]), indicating that higher DTI-ALPS predicted better WM performance through higher Sub-SFC in this subgroup. **C) Mediation model in the higher-PSQI subgroup.** In participants with higher PSQI scores, the indirect effect via Sub-SFC was not significant (*β* = 0.001, SE = 0.026, bootstrapped 95% CI [–0.052, 0.056]). This result is consistent with the absence of a reliable association between Sub-SFC and WM in this subgroup, suggesting that this coupling-related pathway may be attenuated or disrupted when sleep disturbance is more severe.


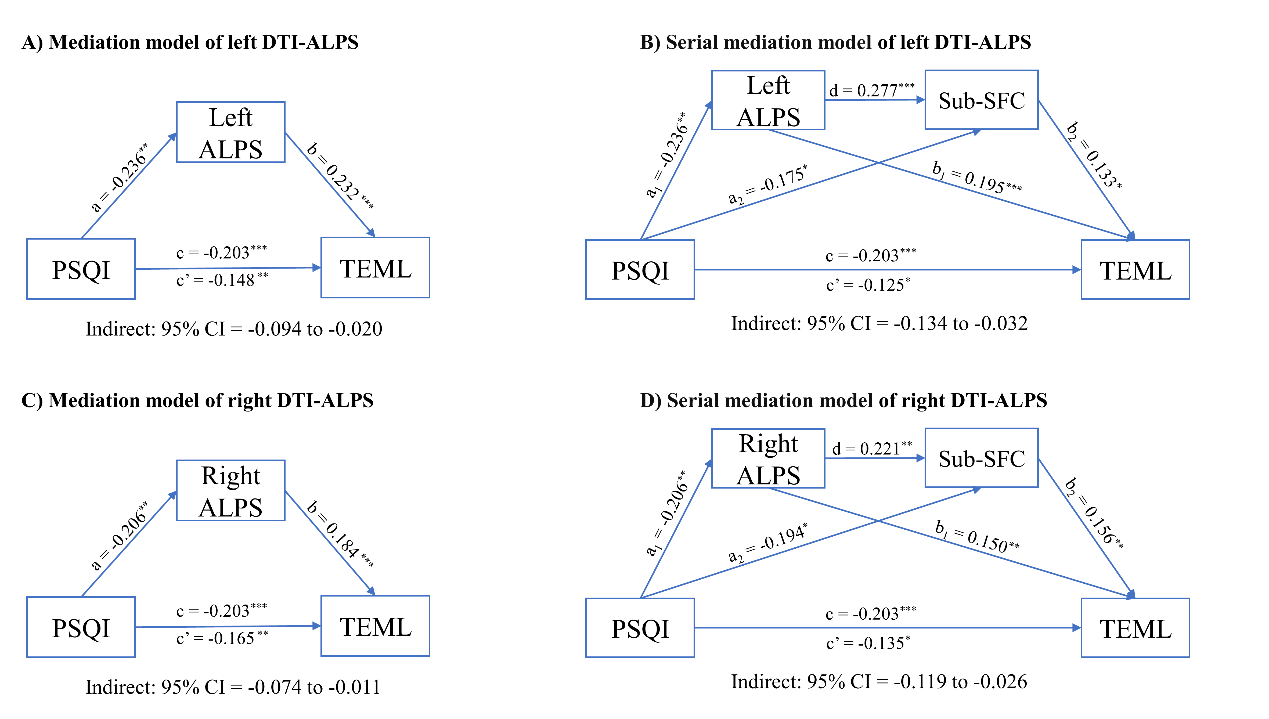


**Figure S6. Mediation and serial mediation models using hemisphere-specific DTI-ALPS indices.** **(A)** Mediation model with left DTI-ALPS as the mediator. **(B)** Serial mediation model with left DTI-ALPS and Sub-SFC. **(C)** Mediation model with right DTI-ALPS as the mediator. **(D)** Serial mediation model with right DTI-ALPS and Sub-SFC.

Abbreviations: PSQI: Pittsburgh Sleep Quality Index; SFC: structural–functional coupling; DTI-ALPS: diffusion tensor image analysis along the perivascular space; TEML: two-error maximum length in work memory task; Sub: subcortical network.

Reference

Cui, Z., Zhong, S., Xu, P., He, Y., & Gong, G. (2013). PANDA: a pipeline toolbox for analyzing brain diffusion images. *Front Hum Neurosci, 7*, 42. doi:10.3389/fnhum.2013.00042

Fan, L., Li, H., Zhuo, J., Zhang, Y., Wang, J., Chen, L., . . . Jiang, T. (2016). The Human Brainnetome Atlas: A New Brain Atlas Based on Connectional Architecture. *Cereb Cortex, 26*(8), 3508-3526. doi:10.1093/cercor/bhw157

Friston, K. J., Williams, S., Howard, R., Frackowiak, R. S., & Turner, R. (1996). Movement-related effects in fMRI time-series. *Magn Reson Med, 35*(3), 346-355. doi:10.1002/mrm.1910350312

Yeo, B. T., Krienen, F. M., Sepulcre, J., Sabuncu, M. R., Lashkari, D., Hollinshead, M., . . . Buckner, R. L. (2011). The organization of the human cerebral cortex estimated by intrinsic functional connectivity. *J Neurophysiol, 106*(3), 1125-1165. doi:10.1152/jn.00338.2011
